# The Antimicrobial Effect of Various Single-Strain and Multi-Strain Probiotics, Dietary Supplements or Other Beneficial Microbes against Common Clinical Wound Pathogens

**DOI:** 10.3390/microorganisms10122518

**Published:** 2022-12-19

**Authors:** Sabina Fijan, Primož Kocbek, Andrej Steyer, Polona Maver Vodičar, Maja Strauss

**Affiliations:** 1 Faculty of Health Sciences, University of Maribor, Žitna ulica 15, 2000 Maribor, Slovenia; 2 Environment and Food, Division of Public Health Microbiology, National Laboratory of Health, Grablovičeva 44, 1000 Ljubljana, Slovenia; 3 Institute of Microbiology and Immunology, Faculty of Medicine, University of Ljubljana, Zaloška 4, 1000 Ljubljana, Slovenia

**Keywords:** probiotics, beneficial microbes, wound pathogens, skin pathogens, agar spot, co-culturing, agar well diffusion, molecular methods, PCR

## Abstract

The skin is the largest organ in the human body and is colonized by a diverse microbiota that works in harmony to protect the skin. However, when skin damage occurs, the skin microbiota is also disrupted, and pathogens can invade the wound and cause infection. Probiotics or other beneficial microbes and their metabolites are one possible alternative treatment for combating skin pathogens via their antimicrobial effectiveness. The objective of our study was to evaluate the antimicrobial effect of seven multi-strain dietary supplements and eleven single-strain microbes that contain probiotics against 15 clinical wound pathogens using the agar spot assay, co-culturing assay, and agar well diffusion assay. We also conducted genera-specific and species-specific molecular methods to detect the DNA in the dietary supplements and single-strain beneficial microbes. We found that the multi-strain dietary supplements exhibited a statistically significant higher antagonistic effect against the challenge wound pathogens than the single-strain microbes and that lactobacilli-containing dietary supplements and single-strain microbes were significantly more efficient than the selected propionibacteria and bacilli. Differences in results between methods were also observed, possibly due to different mechanisms of action. Individual pathogens were susceptible to different dietary supplements or single-strain microbes. Perhaps an individual approach such as a ‘probiogram’ could be a possibility in the future as a method to find the most efficient targeted probiotic strains, cell-free supernatants, or neutralized cell-free supernatants that have the highest antagonistic effect against individual clinical wound pathogens.

## 1. Introduction

The skin is the largest organ in the human body and is colonized by diverse microbiota. Most of these microbes are harmless or even beneficial and serve as physical barriers, protecting our bodies from potential assaults by foreign organisms or toxic substances. The skin, therefore, prevents disruption of this balance caused by the invasion of pathogens due to skin damage because of illness, surgery, and burns [1,2]. Skin damage can be caused by a variety of different reasons such as trauma (including cuts, abrasions, chemical burns, fire burns, cold, heat, radiation, and surgery), or as a consequence of underlying illnesses such as diabetes [3]. The most common wound pathogens include biofilm-forming bacteria such as *Staphylococcus aureus*, *Pseudomonas aeruginosa*, *Enterococcus faecalis*, *Acinetobacter baumannii*, *Escherichia coli*, *Klebsiella pneumoniae*, *Enterobacter* spp., *Bacteroides* spp., *Peptostreptococcus* spp. [4,5,6,7]. Especially chronic wounds are a prominent health concern as they represent an important cause of morbidity and mortality and can significantly reduce the quality of life of patients due to delayed healing, inflammation process, and excessive scarring. They also result in enormous healthcare expenditures [6,8,9,10]. Wound debridement and the topical application of antibiotics or other antimicrobial substances are the conventional methods usually considered to eradicate wound infection. The main disadvantage of recurrent antibiotics used in the context of delayed wound healing and frequent hospitalizations is exacerbated by the rising risk of therapeutic resistance [3].

Probiotics that are by definition ”live microorganisms that, when administered in adequate amounts, confer a health effect on the host” [11] aid in skin healing by stimulating the production of immune cells. They also exhibit antagonistic effects against pathogens via the competitive exclusion of pathogens [3,9,12]. Interestingly enough, the Organization for Economic Cooperation and Development (OECD) also states that probiotics are a promising alternative therapy to the topical use of antibiotics due to the increasing occurrence and transmission of antibiotic-resistant microorganisms [13]. A recent review found that exogenous and oral application of probiotics has shown a reduction in wound infections, especially when used as an adjuvant to antibiotic therapy, and therefore the potential use of probiotics in this field remains worthy of further studies, perhaps focused more on typical skin inhabitants as next-generation probiotics with high potential [9]. On the other hand, using postbiotics could be a safer adjuvant therapy for wound or skin infections as this would mean a safer version of applying metabolites of beneficial microbes without live cells as postbiotics are by definition a “preparation of inanimate microorganisms and/or their components that confers a health benefit on the host” [14].

Some probiotic strains or their cell-free supernatants, mainly from the lactobacilli group, which was recently divided into several genera [15], have shown strong antimicrobial potential against some common wound pathogens using in vitro studies [9]. The investigated probiotics include *Lactiplantibacillus plantarum* ATCC 10241 [16,17], *Limosilactobacillus fermentum* NCIMB 7230 [18], *Limosilactobacillus reuteri* SD2112 [19], *Lacticaseibacillus rhamnosus* GG [20], *Cutibacterium acnes* ATCC 6919 (previously known as *Propionibacterium acnes*) [21] as well as some multi-strain probiotics [22,23,24,25,26] and the investigated pathogens in these studies mainly include *Pseudomonas aeruginosa, Staphylococcus aureus,* and *Escherichia coli*. Animal studies have also shown that topical application of probiotics such as: *Lactiplantibacillus plantarum* ATCC 10241 [17,27,28,29], ATCC 8014 [30], USM8613 [31], *Limosilactobacillus fermentum* NCIMB 7230 [32], and *Cutibacterium acnes* ATCC 6919 [21] were efficient in reducing the pathogen load of skin wounds. The most important type of study to ascertain the efficacy of probiotics is clinical study. In fact, probiotics can only be named as such, if a beneficial effect is supported by at least one well-designed human clinical study [33]. Two human clinical studies have shown that topical application of probiotics reduced pathogen load [5,34] and some recent clinical studies have shown that probiotic consumption indirectly reduced pathogen load via improvement of immune function [9,35,36,37,38,39]. A recent study [40] also addressed the differences between the in vitro and in vivo effects of probiotics on the removal of pathogens using *Lactiplantibacillus plantarum* ATCC 8014 (PTCC 1058) in simulated wound fluid together with *Pseudomonas aeruginosa* and *Staphylococcus aureus* on and animal model. The authors found that the efficacy of probiotics in the presence of different wound pathogens was different and that further investigations are warranted.

To our knowledge, no study has investigated a wide range of single-strain and multiple-strain dietary supplements against a wide range of clinical wound pathogens. Therefore, the aim of our study was to evaluate the antimicrobial effect of eleven single-strain and seven multiple-strain probiotic dietary supplements or other beneficial microbes and their efficiency against fifteen clinical wound pathogens using three methods: agar-spot assay, co-culturing assay, and an agar well diffusion assay, and to statistically compare all results.

## 2. Materials and Methods

### 2.1. Microbial Strains and Clinical Isolates

Eleven single-strain and seven multi-strain dietary supplements or other beneficial microbes noted in Table 1 and Table 2 were used.

As noted in Table 1 and Table 2, all multi-strain dietary supplements MS1 to MS7 and single-strain supplements SS1 to SS4 contain different strains of lactobacilli. Eight strains of the modified *Lactobacillus* genus (MS1 to MS7), seven strains of the *Lacticaseibacillus* genus (MS1, MS2, MS3, MS4, MS5, MS7, SS01, SS02), three strains of *Ligilactobacillus salivarius* (MS1, MS2, MS3, MS5, MS7), three strains of *Lactiplantibacillus plantarum* (MS2, MS6, MS7, SS04), one strain of *Levilactobacillus brevis* (MS1, MS5) and one strain of *Limosilactobacillus reuteri* in SS03. The bifidobacteria genus is included in eight samples: MS1, MS2, MS3, MS5, MS6, MS7, SS05, and SS06. All eight samples include strains of the species *Bifidobacterium animalis* that contains two subspecies: *B. animalis* subsp. *lactis* and *B. animalis* subsp. *animalis.* Two strains of *Bifidobacterium breve* W25, PXN 25 and two strains of *Bifidobacterium longum* W108, PXN 30 in MS5, and MS7, one strain of *Bifidobacterium bifidum* W23 in MS1, MS2, MS5, and MS7 and one strain of *Bifidobacterium infantis* in MS7. Three *lactococci* strains were included in MS1, MS2, MS3, MS5, and MS7. Three propionibacteria strains are included in SS08, SS09, and SS10, [41]. One strain of each of the following bacteria are also included: *Enterococcus faecium* in MS3, *Bacillus subtilis* PXN 21 in MS7, *Bacillus coagulans* MTCC 5260 in SS07, and *Streptococcus thermophilus* in PXN 66 MS7. *Saccharomyces cerevisiae* var. *boulardii* is included in MS6 and SS11. The clinical pathogens were selected from the bacterial strains isolated from the wound samples received at the Institute of Microbiology and immunology at the Faculty of Medicine, University of Ljubljana, Slovenia in 2021. The genera/or species and origin are noted in Table 3.

Clinical strains *Staphylococcus aureus, Pseudomonas aeruginosa, Enterococcus faecalis, Escherichia coli, Klebsiella pneumoniae, Enterobacter* spp., *Acinetobacter* spp. and *Bacteroides* spp. noted in Table 3 were collected from various different skin or wound infections, including skin ulcers, diabetic ulcers, pressure ulcers, inguinal infections, infections at jejunostomy, infections at central venous catheterization, sternal infection, wound dehiscence, surgical wound infection, perianal infection. All pathogens were identified using conventional microbiological methods in the medical diagnostics laboratory Institute of Microbiology and Immunology at the Faculty of Medicine, Ljubljana, Slovenia. All pathogens and probiotics except for those containing lactobacilli were cultured in tryptic soy broth (Fluka, 51228) as overnight cultures, incubated at 35 °C. All lactobacilli containing probiotics or other beneficial microbes were cultured in De Man, Rogosa, and Sharpe broth (Merck, 1.10661) as overnight cultures, incubated at 35 °C.

### 2.2. Molecular Methods for the Detection of Probiotic Strains

For the detection of bacterial and fungal strains of probiotics and other beneficial microbes used in our assays, genus-specific and species-specific PCR primers were used as shown in Table 4.

Bacterial and fungal genomic DNA was extracted from the suspension of microorganisms using PrepMan Ultra Sample Preparation Reagent (Applied Biosystems) in accordance with the manufacturer’s instructions. Amplification was carried out in a thermal cycler (S Labcycler, Sensoquest, Germany), applying the cycling conditions as presented in Table 5. The reaction mixture (50 μL) contained 2.5 U of Taq DNA Polymerase (Qiagen, Germany), 0.5 μM of each primer, 0.2 mM of each deoxyribonucleotide triphosphate, 1.5 mM of 10× reaction buffer, and different concentrations of MgCl_2_ 2.5 mM MgCl_2_ for *Lacticaseibacillus casei*, *Limosilactobacillus reuteri, Lactobacillus gasseri, Lactiplantibacillus plantarum, Bacillus subtilis*, 2 mM MgCl_2_ for *Lacticaseibacillus rhamnosus*, *Lactobacillus acidophilus*, *Bifidobacterium bifidum, Bifidobacterium longum*, 1.5 mM MgCl_2_ for *Bifidobacterium animalis, Bacillus coagulans, Lactococcus* genus, *Saccharomyces cerevisiae*, 1 mM MgCl_2_ for *Propionibacterium* genus, and *Enterococcus faecium* and approx. 10 to 100 ng of bacterial or fungal DNA. In the case of single strains, a lower concentration of template was used to avoid inhibition of the reaction.

Aliquots of the amplified products were subjected to electrophoresis (100 V, 45 min) in 1.5% agarose gel in TBE buffer (89 mM Tris base, 89 mM boric acid, 2 mM EDTA, pH 8.0). Gels were stained with 8 μL of Syber Green I and visualized under UV light (312 nm).

### 2.3. Agar Spot Assay

The antimicrobial effect of the chosen single-strain and multi-strain dietary supplements, probiotics, or other beneficial microbes against common skin or wound pathogens was determined using the modified agar spot assay [55,56,57]. Briefly, each probiotic overnight culture with a final concentration (10^8^ cfu/mL) was inoculated as spots onto the following media: De Man, Rogosa, and Sharpe agar (Millipore, 1.10660) for all multi-strain probiotics and SS01-SS04, TOS-propionate agar (Sigma-Aldrich, 43314) for SS05 and SS06, Mannitol Egg Yolk Polymyxin agar (Merck, 1.05267) for SS07, Clostridium perfrigens agar (Liofilchen, 610207) for SS08-SS10, and Sabouraud glucose agar (BioMerieux, AEB152202) for SS11.

**Figure 1:**
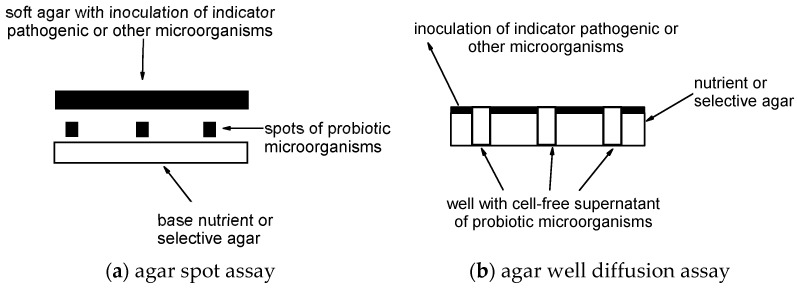
Scheme of the agar spot assay and the agar well diffusion assay. Adapted from Fijan, 2016 [59].

The plates were dried for 30 min at room temperature. All De Man, Rogosa, and Sharpe agar plates were then incubated anaerobically at 35 °C for 24 h using anaerobic jars together with a Genbag anaerobic pouch. Other agar plates were incubated aerobically for 24 h. All plates were then overlaid with 7 mL of soft agar (15g tryptic soy bujon (Fluka, 51228)/500 mL and 2g agarose (Fluka, 51228)/500 mL) inoculated with overnight cultures of the pathogens (with final concentration 7 log cfu/mL) and incubated at 35 °C for 48 h. Figure 1a shows a scheme of the agar spot assay.

After 48 h of incubation, measurements of inhibition zones around the colonies were measured using a ruler. The diameter of the zone of inhibition measuring from both sides of the clear zone around the colony was measured. The result also included 6–7 mm of the colony. Zones of more than 20 mm, between 10 and 20 mm, and less than 10 mm were considered as strong (3+), intermediate (2+), and low inhibitions (+), respectively. This is a modified scale [57], similar to those proposed by Davis and Stout [58]. If no zone was detected the result was reported as less than 6 mm. This assay was performed in triplicate. The mean of the zones of inhibition as well as the standard deviation SD were calculated.

### 2.4. Co-Culturing for Microbial Competition Assay

The co-culturing for microbial competition assay of the pathogens and the chosen single-strain and multi-strain dietary supplements, probiotics, or other beneficial microbes was conducted similarly to Tranberg and co-authors [60] as follows: aliquots of 1 mL of an overnight culture of probiotics and 1 mL of the overnight culture of the clinical wound pathogens were inoculated into 500 mL sterile tubes with fresh broth containing 1 mL tryptic soy broth (Fluka, 51228) and 1 mL De Man, Rogosa and Sharpe broth (Merck, 1.10661). As controls, 1 mL overnight cultures of pathogens were grown in 1 mL tryptic soy broth and 1 mL De Man, Rogosa, and Sharpe broth. All samples were incubated overnight at 35 °C for 24 h.

After 24 h of incubation, colonies of surviving pathogens were counted using serial dilutions, ranging from 10^1^ to 10^8^. The following selective media were used for clinical isolates: Baird-Parker agar (Biolife, 4011162) for *Staphylococcus aureus* isolates, cetrimide agar (22470, Fluka) for *Pseudomonas aeruginosa* isolates, kanamycin esculin azide agar (Biolife, 4015522) for *Enterococcus faecium* isolates, violet red bile glucose agar (Fluka, 70189) for *Escherichia coli* and *Enterobacter* spp. isolates, HiCrome Klebsiella selective agar (Fluka, 90925) for *Klebsiella pneumoniae* isolates, MacConkey agar without salt (Sigma Aldrich, 51405) for *Acinetobacter* spp. isolates and bile esculin agar (Sigma Aldrich, 48300) for *Bacteroides* spp. isolates. All selective media were then incubated aerobically at 35 °C for 24 or 48 h according to the manufacturer’s recommendation except for bile esculin agar for *Bacteroides* spp. isolates which were incubated anaerobically at 35 °C for 24 h using anaerobic jars together with a Genbag anaerobic pouch.

The reduction and log step reduction were then calculated as follows:

$$\%R=\frac{cfu_{pa}-cfu_{pa+pro}}{cfu_{pa}}\times 100$$

$$\log_{10}R=log\frac{cfu_{pa+pro}}{cfu_{pa}}$$

where: %R is the percent of reduction of the pathogen, log_10_R is the log step reduction, cfu_pa_ is the cfu of the pathogen after incubation and cfu_pa+pro_ is the cfu of the pathogen after incubation of the pathogen together with the probiotic. A log step reduction of more than six was considered strong inhibition as it corresponds to a 99.9999% reduction in the case of initial concentration of 10^6^ cfu/mL. Between 3 and 6 was considered intermediate inhibition and less than 3 was considered low inhibition. Two separate experiments were conducted, and the average was calculated for each sample.

### 2.5. Agar Well Diffusion Assay

A slightly modified method of the agar well diffusion assay for the inhibition of pathogens by cell-free supernatants of chosen single-strain and multi-strain dietary supplements, probiotics, or other beneficial microbes by Holder and Boyce [61] was used. Briefly, overnight cultures of pathogens were confluently streaked onto Müller Hinton agar (BioLife, 4017402) plates with sterile cotton swabs and the plates were left to dry for 30 min. Wells (5 mm in diameter) were cut using 1000 µL sterile pipette tips. Cell-free supernatants of overnight cultures of chosen probiotics and other beneficial microbes were prepared by sedimentation of cells with centrifuging (4000× *g* for 10 min). The cell-free supernatant was filtered through a 0.22 µm pore size syringe filter. Half of each cell-free supernatant was used directly by inoculating 800 µL into the wells. The other half was neutralized using NaOH and adjusted to pH = 7 to achieve a neutralized cell-free supernatant that was inoculated into the remaining wells. Figure 1b shows a scheme of the agar well diffusion assay.

The antibacterial effect was determined by measuring the diameter of the zone of inhibition around the wells. Again, zones of more than 20 mm, between 11 and 20 mm, and less than 10 mm were considered strong (3+), intermediate (2+), and low inhibitions (+), respectively. The mean of the radii measuring from the edges of the colonies to the edges of the clear zones was calculated as well as the standard deviation SD. This assay was also performed in triplicate.

After 48 h of incubation, measurements of inhibition zones around the wells were measured using a ruler. The diameter of the zone of inhibition measuring from both sides of the clear zone around the well was measured. The result also included 6 mm diameters of the wells. Zones of more than 20 mm, between 10 and 20 mm, and less than 10 mm were considered as strong (3+), intermediate (2+), and low inhibitions (+), respectively according to the modified scale by Shokryazdan and co-authors [57]. If no zone was detected, the result was reported as less than 6 mm. This assay was performed in triplicate. The mean of the zones of inhibition as well as the standard deviation SD were calculated.

### 2.6. Statistics

The mean zones of inhibition were presented as 95% Confidence Intervals (CI) comparing, agar spot assay, co-culturing, and agar well diffusion assay respectively, explored and evaluated with appropriate statistical as needed for various probiotics groupings, such as single-/multi-strain, species. Student *t*-test was used to compare single-/multi-strains. One-way ANOVA test with post-hoc HSD comparing mean zone was used for multiple probiotic groups and two-factor ANOVA was used to compare agar well diffusion assay interaction with various groups. Assumptions of those tests were also checked. The statistical analysis was performed in the statistical program R (version 4.2.1).

## 3. Results

### 3.1. Identification of Species and Genera of Microbial Strains Using Molecular Detection Methods

The results of the polymerase chain reactions (PCR) using genera-specific and species-specific primer pairs for multi-strain probiotic dietary supplements and single-strain probiotics and other beneficial microbes are noted in Table 6 and Table 7, respectively.

The PCR primer pairs LbLMA1-rev and R-16-1 that targets the nucleotide sequence of the spacer between the 16S and 23S rRNA genes in all lactobacilli genera confirmed by a positive band at 220 bp was found for all seven multi-strain probiotics and all single-strain samples that contained lactobacilli (SS1 to SS4). Species-specific PCR using primer pairs noted in Table 4 was run for the following lactobacilli species: *Lactobacillus acidophilus, Lactobacillus gasseri, Lacticaseibacillus casei, Lactocaseibacillus rhamnosus, Lactiplantibacillus plantarum* and *Limosilactobacillus reuteri*. Species-specific DNA fragments were found for *Lactobacillus acidophilus* in MS1, MS2, MS3, MS5, MS6, MS7, *Lactobacillus gasseri* in MS4, *Lacticaseibacillus casei* in MS1, MS2, MS3, MS5, MS7, and SS02, *Lactocaseibacillus rhamnosus* in MS4, MS7, and SS01, *Lactiplantibacillus plantarum* in MS2, MS6, MS7, and SS04 and *Limosilactobacillus reuteri* in SS03.

The genus *Bifidobacterium* using the primer pairs Bif164F and Bif601R for amplifying the 16S ribosomal rRNA fragments confirmed by a positive band at 453 bp was also confirmed for all bifidobacterial containing samples (MS1, MS2, MS3, MS5, MS6, MS7, SS05, SS06). Species-specific PCR using primer pairs noted in Table 4 was run for the following bifidobacterial species: *Bifidobacterium animalis*, *Bifidobacterium bifidum*, and *Bifidobacterium longum.* Species-specific DNA fragments were found for *Bifidobacterium animalis* in MS1, MS2, MS3, MS5, MS6, MS7, SS5, and SS6, *Bifidobacterium bifidum* in MS1, MS2, MS5, and MS7 and *Bifidobacterium longum* in MS5 and MS7. The genera *Lactococcus* and *Propionibacterium* were confirmed by primer pairs noted in Table 4 for MS1, MS2, MS3, MS5, and MS7 and SS08, as well as SS09 and SS10, respectively. Species-specific DNA fragments were also found for the bacteria *Enterococcus faecium* (MS3), *Bacillus subtilis* (MS7), and *Bacillus coagulans* (SS07). *Saccharomyces cerevisiae* species-specific DNA fragments were found (MS6, SS11) thus confirming the presence of *Saccharomyces cerevisiae* subsp. *boulardii*.

### 3.2. Agar Spot Assay

The evaluation of the mean zone of inhibition and standard deviation of the agar spot assay for all investigated probiotics and other beneficial microbes against the clinical pathogens, isolated from various skin wounds are noted in Table 8. All results of the zone of inhibition and standard deviation are noted in Supplementary Table S1.

As obvious from Table 8, all multiple-strain probiotics and single-strain probiotics SS01to SS04 (including *Lacticaseibacillus paracasei* Shirota, *Limosilactobacillus reuteri* DSM 17938, *Lacticaseibacillus rhamnosus* GG, and *Lactiplantibacillus plantarum* DSM 2601) were successful against most clinical wound pathogens as strong inhibition (the zone of inhibition was more than 20 mm) was found in most of the assays. On the other hand, the single strain probiotics *Bacillus coagulans* MTCC 5260 (SS07), *Propionibacterium freudenreichii* DSM 20271 (SS08), *Propionibacterium propionici* DSM 20272 (SS09), and *Propionibacterium freudenreichii* susp. *shermanii* (SS10) exhibited only low inhibition (the zone of inhibition was less than 10 mm). Intermediate average inhibition (zone of inhibition was between 10 and 20 mm) was found for both single-strain bifidobacteria: *Bifidobacterium lactis* HN019 (SS05), *Bifidobacterium lactis* BB12 (SS06), and the single-strain fungi *Saccharomyces boulardii* (SS11). The average zone of inhibition of all probiotic strains against individual clinical pathogens was intermediate for most strains and even above 20 mm for one strain of *Staphylococcus aureus*, *Pseudomonas aeruginosa*, *Escherichia coli*, and *Acinetobacter* and both clinical strains of the *Bacteroides* genus thus indicating that no specific pathogen stood out or was more resistant to the antimicrobial effect of the chosen probiotics.

**Figure 2:**
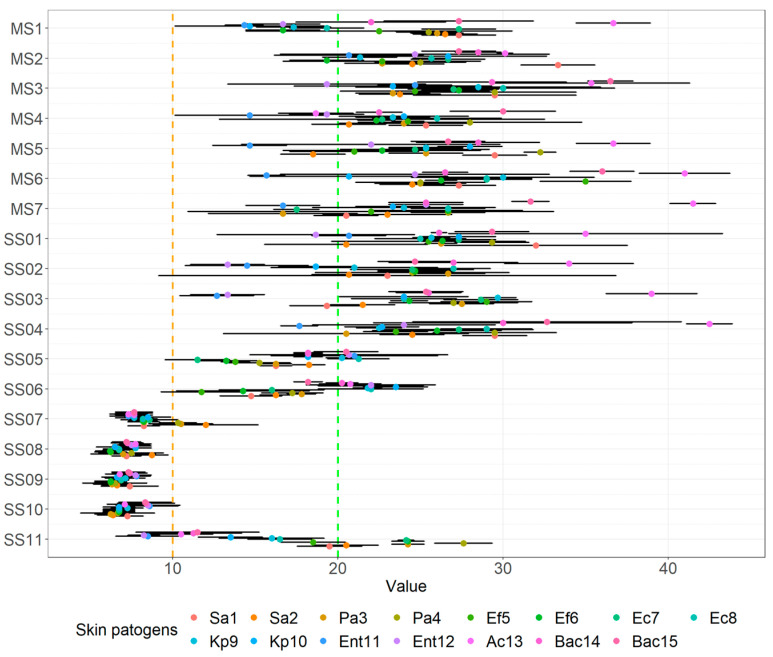
Means of inhibition zone together with 95% CI of various probiotics and other beneficial microbes against clinical skin pathogens using the agar spot assay.

The means of the inhibition zone of probiotics against wound pathogens with 95% CI are noted in Figure 2. The *Propionibacterium* strains and the *Bacillus* strain (SS07-SS10) seem to have smaller mean zones of inhibition and all multi-strain probiotics seem to have a larger zone of inhibition against all challenge wound pathogens. Checking the mean zone of inhibition against all wound pathogens for the various probiotics we observed statistical differences (F(17.252) = 40.5, *p* < 0.001).

**Figure 3:**
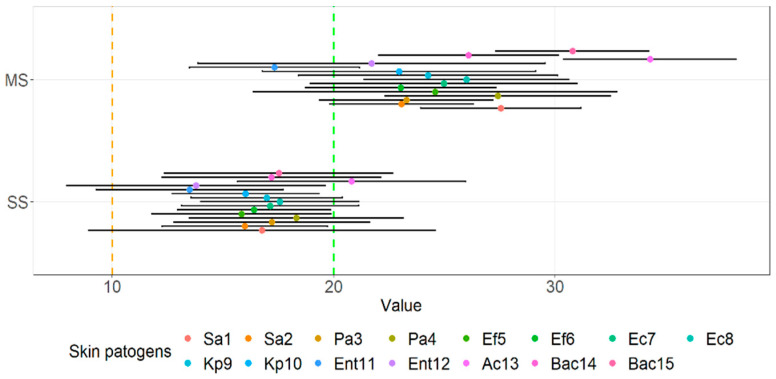
Means of inhibition zone together with 95% CI of multi-strain and single-strain probiotics against clinical skin pathogens using the agar spot assay.

As obvious in Figure 3, looking just at multi-strain (M = 25.15, SD = 3.95) and single-strain probiotics or beneficial microbes (M = 16.74, SD = 1.74), we showed that the latter has a statistically significant lower mean inhibition zone (t = −7.553, *p* < 0.001), which is also indicated in Figure 3. Grouping the data along the lines for species we observed the average means of the inhibition zone in descending order as follows: multi-strain probiotics that contained mainly lactobacilli strains and single-strain lactobacilli (M = 25.20, SD = 4.20), single strain bifidobacteria probiotics (M = 18.05, SD = 3.09), probiotic yeast strain and *Bacillus* strain (M = 16.98, SD = 6.31), and finally the *Propionibacterium* (M = 7.48, SD = 0.42). These means had a statistically significant difference (F(3.56) = 47.38, *p* < 0.001).

**Figure 4:**
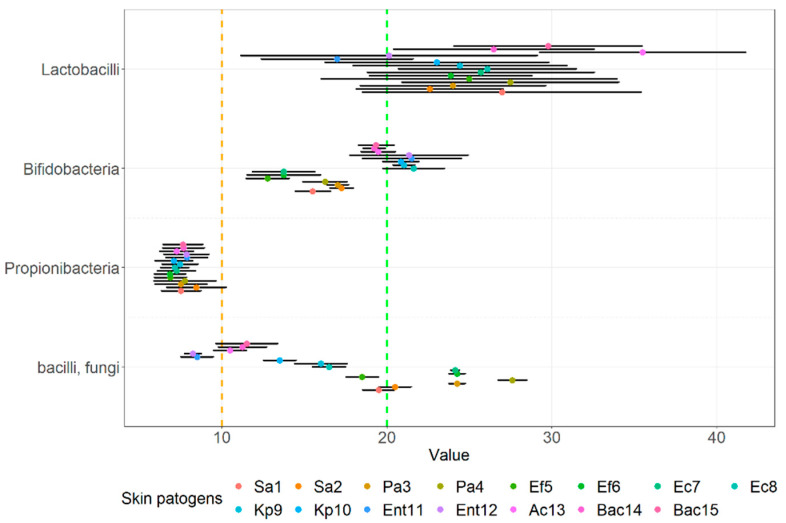
Means of inhibition zones together with 95% CI of probiotics and other beneficial microbes, divided into groups according to main species, against clinical skin pathogens using the agar spot assay.

A post-hoc HSD test comparing pairs showed that the mean zone of inhibition against wound pathogens for all lactobacilli-containing probiotics was higher than others and the mean zone of inhibition of the *Propionibacterium* strains was lower than the others, which can also be at least partially indicated in Figure 4.

### 3.3. Co-Culturing for Microbial Competition Assay

The evaluation of the average log step reduction for all investigated probiotics and other beneficial microbes against the clinical pathogens, isolated from various skin wounds using co-culturing is noted in Table 9. The scale of a log step reduction of more than 6 was considered strong inhibition, between 3 and 6 was considered intermediate inhibition and less than 3 was considered low inhibition. All results of the average log step reduction and percentage of reduction are noted in Supplementary Table S2.

As obvious from Table 9 strong reduction of pathogens (log step reduction of more than 6 log steps) was found for three multiple-strain probiotics (MS4, MS5, and MS7), whilst a low reduction of pathogens was found for three single-strain probiotics including *Propionibacterium freudenreichii* DSM 20271, *Propionibacterium propionici* DSM 20272, and *Saccharomyces boulardii* (SS8, SS9, and SS11 respectively). All other probiotics achieved an intermediate reduction of pathogens (log step reduction between 3 and 6 log steps). One clinical pathogen of the *Enterobacter* genus was less resistant as an average log step reduction above 6 log steps was achieved for all probiotics and one clinical pathogen of *Enterococcus faecalis* was most resistant as the average log step reduction under 3 log steps was achieved for all probiotics. Comparing the log step reduction of the wound pathogens after co-culturing with probiotics and other beneficial microbes we observed lower inhibition compared to agar spot assays for all probiotics and other beneficial microbes (F(17.252) = 12.08, *p* < 0.001). 

The same was observed when comparing the inhibition effect of multi-strain and single-strain probiotics against wound pathogens (t = −3.962, *p* < 0.001), where multi-strain probiotics (M = 5.62, SD = 129) achieved a higher log step reduction of all challenge pathogens than single-strain probiotics (M = 3.94, SD = 1.03). When comparing the inhibition effect of probiotic species against all pathogens we found a statistical difference (F(3.56) = 26.79, *p* < 0.001). When comparing pairs with HSD post-hoc tests, we showed that there was no statistical difference between the inhibition effect of lactobacilli-containing probiotics (M = 5.30, SD = 1.20) and bifidobacteria-containing probiotics (M = 5.51, SD = 1.38) against the wound pathogens. However, there was a difference when comparing both lactobacilli and bifidobacteria containing probiotics to single-strain beneficial microbes that contained propionibacteria, the *Bacillus* species, and the probiotic yeast. Additionally, there was no difference between the probiotic yeast (M = 2.25, SD = 1.46) and the beneficial microbes that contained propionibacteria (M = 2.78, SD = 0.94).

### 3.4. Agar Well Diffusion Assay

Below are the results of the mean zone of inhibition for all investigated cell-free supernatants (S) (Table 10) and neutralized cell-free supernatants (NS) (Table 10) of probiotics and other beneficial microbes against the clinical pathogens, isolated from various skin wounds. Exact values of inhibition zones and standard deviation are noted in Supplementary Tables S3 and S4.

As obvious from Table 10, the cell-free supernatants of all multiple-strain probiotics except MS7 exhibited an intermediate average inhibition (zone of inhibition was between 10 and 20 mm). All cell-free supernatants of single-strain probiotics and MS7 exhibited only a low inhibition (the zone of inhibition was less than 10 mm). Only two probiotics (MS6 and SS01) exhibited high inhibition of cell-free supernatant, both for the same clinical strain of *Escherichia coli*. No pathogen stood out in its resistance against the cell-free supernatants. All results show a lower inhibition ability of the cell-free supernatant compared to probiotics.

As obvious from Table 11 (Supplementary Table S4) the neutralized cell-free supernatants of all probiotic strains exhibited only low average inhibition for all investigated clinical pathogens from wounds. Only one neutralized cell-free supernatant of *Limosilactobacillus reuteri* DSM 17938 exhibited a strong inhibition against one clinical strain from the *Bacteroides* genus. No pathogen stood out in its resistance against the neutralized cell-free supernatants. All results show a lower ability of the neutralized cell-free supernatant compared to cell-free supernatants.

**Figure 5:**
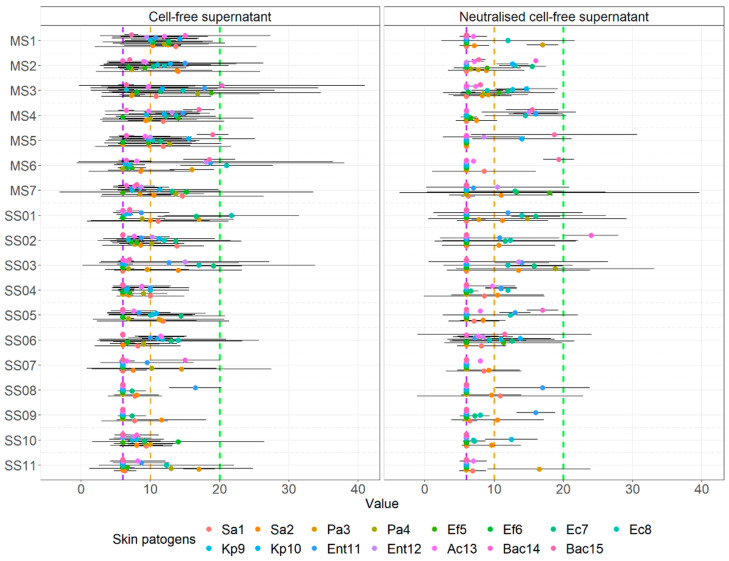
Means of inhibition zone together with 95% CI of various cell-free supernatants of probiotics and other beneficial microbes against clinical skin pathogens using the agar well diffusion assay.

**Figure 6:**
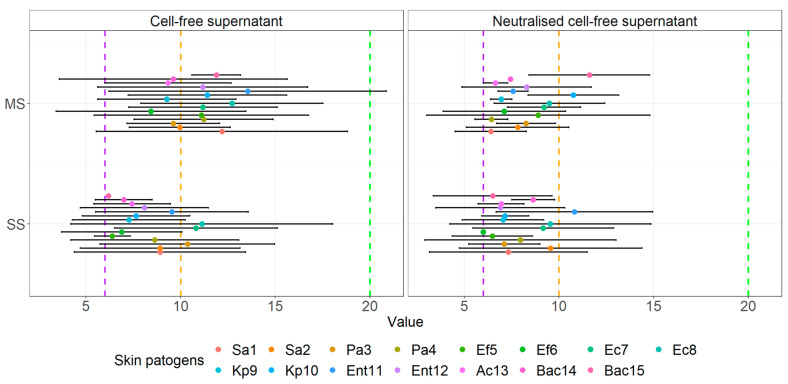
Means of the inhibition zone together with 95% CI of cell-free supernatants of multi-strain and single-strain probiotics against clinical skin pathogens using the agar well diffusion assay.

The visual comparison of the results of the inhibition zones of cell-free supernatants (S) and neutralized cell-free supernatants (NS) of all probiotics and beneficial microbes against clinical pathogens are noted in Figure 5. Figure 6 displays the comparison of the inhibition zones of S and NS of multi- and single- strain probiotics and microbes against clinical wound pathogens and Figure 6 displays the results of the inhibition zones of S and NS for all probiotics and beneficial microbes, divides into main species against the wound pathogens.

Statistically comparing results of the inhibition of cell-free supernatant and neutralized cell-free supernatants agar well diffusion, S and NS respectfully (Figure 6), with the aforementioned groups we observed, that there was no statistically significant interaction between agar well diffusion results and all probiotics of beneficial microbes (F(17.504) = 1.281, *p =* 0.199), but there was a simple main effect on various probiotics (*p* < 0.001) and agar well diffusion (*p* < 0.001) on the mean zone.

**Figure 7:**
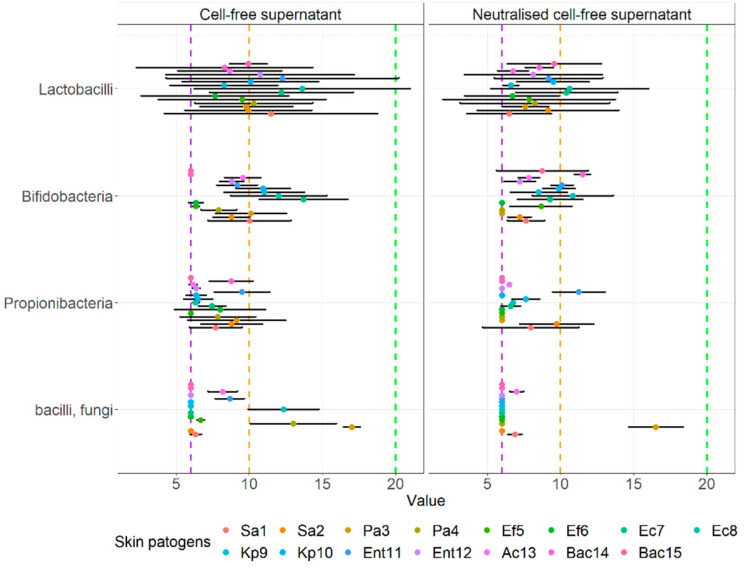
Means of inhibition zone together with 95% CI of cell-free supernatants of probiotics and other beneficial microbes, divided into groups according to main species, against clinical skin pathogens using the agar well diffusion assay.

Looking at multi- and single- strain probiotics (Figure 7), we can observe a statistically significant interaction between the effect of both S and N supernatants using the agar well diffusion assay against wound pathogens (F(1.56) = 7.475, *p =* 0.008) as well as simple main effects, more precisely mean zones of supernatants of multi-stain probiotics were higher compared to single-stain supernatants (*p* < 0.001) and inhibition was higher for S compared to NS (*p* < 0.001). There was also no interaction between the inhibition of cell-free supernatants of probiotics and other beneficial microbes, divided into main species (F(3.112) = 2.740, *p =* 0.610) against all wound pathogens. However, both supernatants exhibited significant simple main effects, higher for S than NS (*p =* 0.007) and also higher for bifidobacteria-containing single strain probiotics (*p =* 0.030) and lactobacilli-containing probiotics (*p =* 0.001) than for propionibacteria-containing single strain beneficial microbes (*p =* 0.005).

## 4. Discussion

One important attribute of probiotics and probiotic candidates is their antimicrobial effect against pathogens. It is a well-known attribute of the lactobacilli and bifidobacteria genera [62]. The antimicrobial effect against pathogens is mostly attributed to the production of metabolites such as bacteriocins, organic acids, short-chain fatty acids, and hydrogen peroxide. Other important mechanisms of action of probiotics include competitive exclusion, immune modulation, stimulation of host defenses, and the production of signaling molecules that trigger changes in gene expression [55,63,64]. However, appropriate methodology is important in order to determine realistic and repeatable results. Our study used three different in vitro methods for determining the antimicrobial effect: the agar-spot assay, the co-culturing assay, and the agar-well diffusion assay. The first two methods utilised live microbes, whilst the last method utilised cell-free supernatant or postbiotics. The methods presented differences in the results. All dietary supplements achieved a certain level of inhibition of all pathogens, although there were variations between strains and multi-strain supplements, where the latter exhibited higher inhibition of the clinical pathogens than the single strain (*p* < 0.05), regardless of the method.

Our analysis of the collected data showed that the means of inhibition of probiotics and other beneficial microbes against all wound pathogens were statistically different (F(17.252) = 40.5, *p* < 0.001), where single strain beneficial microbes containing propionibacteria and *Bacillus* species (SS07-SS10) exhibited smaller inhibition zones against wound pathogens compared to all other probiotics and all multi-strain probiotics exhibited larger inhibition than single-strain probiotics. Looking at interactions via two-way ANOVA analysis, we observed a statistically significant interaction between multi- and single- strain probiotics or beneficial microbes and agar well diffusion (F(1.56) = 7.475, *p =* 0.008) as well as higher simple main effects for mean zones of multi-stain probiotics compared to single-stains (*p* < 0.001) and mean zones in agar well diffusion was lower in neutralized supernatant compared to the supernatant (*p* < 0.001) against wound pathogens.

The three methods to assess the antimicrobial effect of probiotics and other beneficial microbes or their metabolites against skin pathogens deployed in this study are based on phenotype characteristics that can be used for culturable microorganisms [65]. The most time-consuming is the co-culturing assay which requires the preparation of 10-fold dilutions for the enumeration of the pathogen after incubation with probiotics to determine the reduction effect. There are several modifications to this method, including incubation time, media type, and final detection method [55,66,67,68]. The advantage of this method is that both the probiotics and the pathogens are in a liquid environment enabling more simulation of the natural environment than existing in a colony on a solid surface, where immobilisation restricts growth. Such an environment causes different dynamics, less growth restriction, quorum sensing, and planktonic growth of both the probiotic and pathogen microbes [69,70,71]. Both the agar-well diffusion assay and the agar spot assay are conducted on solid media and require the measurement of the zone of inhibition against challenge pathogens. The agar spot assay investigates the inhibition effect of microbes, grown in a colony, whilst the agar-well diffusion assay investigates the inhibition effect of the cell-free supernatant, either in direct form or neutralized to eliminate the effect of organic acids. Both methods also exhibit several modifications with regard to solid media preparation, incubation conditions, initial concentration, and diffusion of metabolites [21,22,25,66,72,73,74]. Some authors measured either the whole diameter of the zone of inhibition which includes the diameter of the formed probiotic colony or the well with the supernatant [22,66,72,73], whilst other authors measured only the radius of the inhibition zone [25,55,74]. In our study, all cell-free supernatants of lactobacilli-containing multi-strain dietary supplements exhibited some inhibition as the average inhibition was intermediate for all except MS7. However, the cell-free supernatants of single-strain lactobacilli achieved average low inhibition. In the study by Lopes and co-authors [25], all investigated lactobacilli strains exhibited inhibition against examples of possible wound pathogens including *Escherichia coli, Pseudomonas aeruginosa*, and *Staphylococcus aureus.* However, when examining the results, it is obvious that in some cases, the radius of the zone of inhibition is only 1 mm, meaning the inhibition was also low for some strains, as found in our study. Similarly, in the study by Tejero-Sarinena and co-authors [74] the radii of the zones of inhibition of the non-adjusted cell-free supernatant of various lactobacilli and bifidobacteria strains were low, between 0.7 mm and up to 2 mm.

Neutralization of the culture supernatants with alkali vastly reduced the antagonistic effects of all our multiple-strain dietary supplements and our single strains thus indicating that the main mechanism of antagonism was the production of organic acids, such as lactic acid, propionic acid, butyric acid, and that bacteriocinogenic potentials were only partially used. On the other hand, the neutralized cell-free supernatant of *Lactiplantibacillus plantarum* DSM 2601 (SS04), *Propionibacterium freudenreichii* DSM 20271 (SS08), and *Propionibacterium acidipropionici* DSM 20272 (SS09) exhibited a somewhat higher average inhibition than the non-adapted cell-free supernatant, thus indicating that bacteriocins, such as perhaps plataricins, pediocins, or other neutral metabolites were produced [75,76,77]. The neutralized cell-free supernatant *Bifidobacterium animalis* subsp. *lactis* BB12 had a higher antagonistic effect than the non-neutralized for some pathogens (*Enterococcus faecium, Escherichia coli, Klebsiella pneumoniae,* and *Bacteroides* spp.) but not for *Pseudomonas aeruginosa*, *Enterobacter* spp. or *Acinetobacter* spp. Similarly, in the study by Fredua-Agyeman and co-authors [78] the neutralized cell-free supernatants of BB12 and *Lactobacillus acidophilus* La-5 did not show inhibition against *Pseudomonas aeruginosa*. Additionally, in the study by Lopes and co-authors [25], the antimicrobial activity of the cell-free supernatant was also attributed to organic acid production as the neutralized supernatant did not exhibit inhibition. The same conclusions were also found in the study by Tejero-Sarinena and co-authors [74].

In order to enable some comparison between all three methods, we created a scale of the co-culturing method based on the disinfection requirement for medical devices according to the Food and Drug Administration [79], where a log step reduction of 6 log steps or more is considered a strong reduction of the pathogen. This was based on the disinfection requirement of disinfectants where the log step reduction of 6 log steps corresponds to a 99.9999% reduction in the case of the initial pathogen concentration of 10^6^ cfu. All chosen probiotics achieved a log step reduction for all challenge pathogens. According to the scale, three of our five chosen multi-strain dietary supplements (MS4, MS5, MS7) exhibited a strong average reduction of pathogens, whilst the other multi-strain dietary supplements achieved intermediate log step reduction of the pathogen. Eight of our chosen single-strain beneficial microbes achieved an average intermediate reduction of pathogens, whilst three achieved a low average reduction of the pathogen (*Propionibacterium freudenreichii* DSM 20271, *Propionibacterium acidipropionici* DSM 20272, and *Saccharomyces boulardii*). Other studies using the co-culturing method found that probiotics or probiotic candidates caused a reduction of pathogens, including *Escherichia coli* and *Staphylococcus aureus* co-cultured with *Lactobacillus acidophilus* La5 and *Bifidobacterium longum* ATCC 15707 [67], *Staphylococcus aureus,* and *Pseudomonas aeruginosa* co-cultured with *Limosilactobacillus fermentum* [68], *Escherichia coli*, *Salmonella* Enteritidis, *Salmonella* serotype (ser.) Typhimurium, *Staphylococcus intermedius*, *Klebsiella oxytoca,* and other pathogens co-cultured with lactobacilli isolated from piglet feces [66].

Molecular methods are much less time-consuming than classical phenotype methods that cannot easily distinguish between various species of the same genera and are also applicable for enumeration [80,81]. Although we did not conduct all species-specific PCR protocols for all species declared in all dietary supplements, we found positive results for all the PCR protocols that we conducted to detect genera or species thus proving that the reliability of the labelling system of probiotic supplements has improved compared to previous years [43,82]. Despite the recent division of lactobacilli into 23 novel genera [15] we found positive bands for all lactobacilli-containing dietary supplements using the primers pairs LbMA1-rev/R-16-1 [42], and it is obvious that these new genera share a common DNA section. Almost no dietary supplements used this new nomenclature. Another interesting finding was the positive band for *Lacticaseibacillus paracasei* Shirota (SS01, Yakult^®^), using the primer pairs Prl/CasII for the *casei* species, published by Walter and co-authors in the year 2000 [44]. As *Lacticaseibacillus paracasei* Shirota was reclassified from the *casei* species [83] after the publication of the primers, it is obvious that they are not species-specific and share a common DNA section as they belong to closely related species [84]. Recently the heterogenous genus of propionibacteria was divided into cutaneous (*Cutibacterium* spp.) and dairy propionic acid-producing bacteria (*Propionibacterium* and *Acidipropionibacterium* spp.) [41]; however, using the primer pairs PB1/PB2 [50] all propionibacteria: *Propionibacterium freudenreichii* subsp. *Freudenreichii* DSM 20271, *Propionibacterium freudenreichii* subsp. *Shermanii, and Acidipropionibacterium acidipropionici* DSM 20271 (SS08-SS10, respectively) were detected. Additionally, the primer pair CS1/SC2 [54] was used to detect *Saccharomyces cerevisiae* and we found a positive band for MS6 and SS11, which both contain *Saccharomyces boulardii* according to the manufacturers, confirming it is in fact a variant of *Saccharomyces cerevisiae* [54]. These findings also indicate that all manufacturers are not up to date with taxonomic changes.

The probiotic *Lacticaseibacillus rhamnosus* GG, also known as LGG, was the first lactobacilli strain to be patented in 1989 and has proven health benefits as shown by systematic reviews of several clinical studies, focused on antibiotic-associated diarrhoea [85], paediatric diarrhoea [86], gastroenteritis [87] and respiratory tract infections in children [88]. It is a biofilm-forming and immunomodulating probiotic that has shown antimicrobial effect against several pathogens [89] and is often used in in vitro studies as a reference strain for examining the antimicrobial effect of potential new probiotic strains [90,91]. In our study, this strain was SS03 and it also exhibited strong inhibition of most clinical pathogens using the agar spot method. However, using the co-culturing method, our results show that only an intermediate inhibition rate was achieved, thus implying that complex mechanisms of the probiotic are at work in different circumstances and that promising in vitro results using one method does not necessarily correlate with other methods [55] or correlate to statistically significant health benefits in clinical studies [92,93].

*Lacticaseibacillus paracasei* Shirota and *Limosilactobacillus reuteri* DSM 17938 are also well-researched probiotic strains (SS01 and SS02). Both strains exhibited the same results as SS03, namely strong average inhibition using the agar spot assay and an intermediate inhibition rate using the co-culturing assay. The same results were also found for the less-researched strain *Lactiplantibacillus plantarum* DSM 2601 (SS04). The latest clinical studies of *Lacticaseibacillus paracasei* Shirota (Yakult^®^), find consumption leads to improvement of depressive symptoms [83], lipid metabolism and intestinal microbiota [94], digestive disorders [95], and immunological function [96]. *Lacticaseibacillus paracasei* Shirota has also shown antifungal activity [97] and, similarly to our study, antibacterial activity against *Escherichia coli* and *Bacteroides* spp. [98]. *Limosilactobacillus reuteri* DSM 17938 (BioGaia^®^) has replaced the original strain *Limosilactobacillus reuteri* ATCC 55730 as it does not contain plasmid-borne antibiotic resistance and both strains exhibit success in the treatment of acute gastroenteritis, especially in children [99]. Although *Limosilactobacillus reuteri* DSM 17938 exhibits strong antimicrobial potential against major gastric and enteric bacterial pathogens and rotavirus [100], it did not prove effective as eradication therapy for infection with *Helicobacter pylori*, thus indicating that further studies are needed to establish the role of probiotics as adjuvant therapy, as the authors concluded [101].

Two well-known strains of the same species of bifidobacteria were used as single-strain probiotics: *Bifibacterium animalis* subs. *lactis* HN019 and BB-12 (SS05 and SS06, respectively). Both strains exhibited comparable results using the agar spot assay. On the other hand, there were differences in individual results for the inhibition of pathogens for co-culturing and the agar-well diffusion assay, although the average inhibitions were almost the value, proving that many probiotics traits are indeed strain-specific [11] and cannot be generalized to all representatives of the same species. The strain HN019 proved successful against periodontal pathogens in a recent clinical trial [102] and is a well-known probiotic with immune-enhancing properties [103]. In an in vitro study using the co-culturing method *Bifidobacterium animalis* subs. *Lactis,* BB-12 successfully inhibited the growth of *Clostridoides difficile* (previously known as *Clostridium difficile*) [104]. This strain reduced the risk of respiratory infections in infancy in a clinical study [105].

The challenge propionic acid-producing bacteria used in our study included three strains: *Propionibacterium freudenreichii* subsp. *freudenreichii* DSM 20271, *Acidipropionibacterium acidipropionici* DSM 2072, and *Propionibacterium freudenreichii* subsp. *Shermanii* (SS08, 09, and 10, respectively), and achieved only intermediate, low, or even no average inhibition of pathogens, depending on the method. The common feature of these three bacteria is the ability to produce propionic acid. Our results are similar to the study by Dyshlyuk and co-authors [106] where moderate antimicrobial activity using a version of the agar spot method was found for *Propionibacterium jensenii* B-6085 and *Propionibacterium thoenii* B-6082, but not for *Propionibacterium freudenreichii* B-11921 and *Propionibacterium acidipropionici* B-5723 against pathogens *Escherichia coli* ATCC 25922, *Salmonella enterica* ATCC 14028, *Staphylococcus aureus* ATCC 25923, *Pseudomonas aeruginosa* B6643, *Proteus vulgaris* ATCC 63, and *Listeria monocytogenes* ATCC 7644. *Propionibacterium freudenreichii* subsp. *freudenreichii* DSM 20271 is known to produce cobalamin or vitamin B12 [107] and *Propionibacterium freudenreichii* subsp. *shermanii* has shown probiotic effect as part of multi-strain dietary supplements in clinical studies against irritable bowel syndrome-related intestinal microbiota stabilization [108], intestinal microbiota changes during anti-*Helicobacter pylori* treatment [109].

Our challenge spore-forming representative *Bacillus coagulans* MTCC 5260 (Prolife^®^) also achieved only intermediate, low, or even no average inhibition of pathogens, depending on the method. Probiotic *Bacillus* strains used either in spore or vegetative forms have shown antimicrobial, anticancer, antioxidant, and vitamin production properties. However, they can also produce toxins and biogenic amines and transfer antibiotic resistance genes; therefore, their safety is a concern. Studies on the microbiome using probiotic *Bacillus* strains are limited in humans [110]. The strain MTCC 5260 is also known as Unique IS2 and ATCC PTA-11748 [111] and has documented clinical efficacy against constipation [112]. It also exhibits antimicrobial effectiveness as it was efficient as an adjuvant in the treatment of bacterial vaginosis [113].

*Saccharomyces cerevisiae* var. *boulardii* (SS11) is the only representative of probiotic fungi used in our study and it achieved average intermediate or low pathogen reduction, depending on the method used. It is a well-known probiotic that produces various bioactive compounds and is mostly known for its role in treating gastrointestinal diseases [114,115]. Together with *Lacticaseibacillus rhamnosus* GG, it is even one of the few probiotics recommended by the ESPGHAN (European Society for Paediatric Gastroenterology, Hepatology, and Nutrition) and ESPID (European Society for Paediatric Infectious Diseases) [116,117] for treating acute gastroenteritis in children. *Saccharomyces boulardii* has also been proposed as an alternative to treating bacterial infections [114], however, our results do not support this claim for our challenge wound pathogens.

In our study, the multi-strain dietary supplement MS7 (Bio-Kult^®^) was effective in strong average inhibition found against most clinical pathogens using the agar spot method. This dietary supplement was also the most effective mixture against *Enterococcus faecalis* in another study using the agar spot test [72]. In a clinical study, this multi-strain probiotic was also associated with significant improvement in symptoms in patents with diarrhoea-predominated irritable bowel syndrome [118].

Several commercial dietary supplements including OMNi-BiOTic^®^ Hetox, OMNi-BiOTic^®^ 6, OMNi-BiOTic^®^ Stress repair, OMNi-BiOTic^®^ Flora plus+, and OMNi-BiOTic^®^ Activ (MS1 to MS5) achieved strong average inhibition against all pathogens in our study. MS1, MS4, and MS5 also achieved strong average inhibition using the co-culturing method, whilst the average inhibition of cell-free supernatant was intermediate or even low. The lower effect of cell-free probiotic supernatant indicates that bacterial response is important in cell-cell signaling and/or bacteria-host interaction. The multi-strain dietary supplement MS2 was also used in a clinical study that found that this multi-strain probiotic might be a well-tolerated tool to positively influence the gastrointestinal quality of life as well as mental and somatic health, cognition, and immune response and potentially have effects on psychiatric symptoms [119]. In another clinical study, this multi-strain probiotic positively influenced the gastrointestinal tract of patients with diarrhoea-predominated irritable bowel syndrome [120]. In another study, the multi-strain postbiotic supernatant of the dietary supplement OMNi-BiOTic AAD10 with similar composition exhibited positive antibacterial and antifungal effects in vitro [121].

Our results show that several dietary supplements were efficient in reducing the pathogen loads of the investigated clinical pathogens. The concept that certain bacteria can destroy other, even pathogenic bacteria, especially with respect to the skin, is not new and many historic researchers, such as Metchnikoff, Nissle, Cantini, and others have investigated and proven this concept [9,122,123]. More than a decade ago, Howard and co-authors concluded that probiotics could be beneficial in the prevention and treatment of wound infections [124]. Probiotics also give positive results for wound healing, wound-epithelization, and neovascularization [125]; however, as such treatment represents a shift in the doctrine of wound treatments where using bacteria to fight bacteria is not intuitive [126,127], many more studies are needed to establish a consensus on the efficacy of using probiotics against skin pathogens.

## 5. Conclusions

The scientific evidence of the health benefits of using probiotics and postbiotics for wounds is becoming more extensive and, therefore, an important possible application of probiotics in the future. In light of our results, it seems that each clinical pathogen was differently susceptible to different probiotic strains, although in general the multispecies probiotics were more efficient than the single-strain probiotics; however, the method deployed also impacted the results. Perhaps a new approach such as a ‘’probiogram’’ or ‘’postbiogram’’ as an analogue to antibiograms could be a possibility in the future in finding the most efficient targeted probiotic strains, cell-free supernatants, or neutralized cell-free supernatants that have the highest antagonistic effect against individual clinical wound pathogens. Additionally, more robust, well-designed clinical trials of probiotics targeting different clinical skin pathogens are needed to establish more knowledge on the exact efficacy and mechanisms of individual probiotics against pathogens to draw evidence-based conclusions for clinical recommendations.

## Figures and Tables

**Table 1:** Multi-strain dietary supplements containing various probiotic strains.

Label	Supplement	Lactobacilli ^1^	Bifidobacteria	Other Bacteria or Fungi
MS1	OMNi-BiOTiC^®^ HETOX, Institut Allergosan, Austria	*Lacticaseibacillus casei* W56 *Lactobacillus acidophilus* W37 *Levilactobacillus brevis* W63 *Lactobacillus salivarius* W24	*Bifidobacterium lactis* W52 *Bifidobacterium bifidum* W23	*Lactococcus lactis* W58 *Lactococcus lactis* W19
MS 2	OMNi-BiOTiC^®^ STRESS Repair, Institut Allergosan, Austria	*Lacticaseibacillus casei* W56 *Lactobacillus acidophilus* W22 *Lacticaseibacillus paracasei* W20 *Lactiplantibacillus plantarum* W62 *Ligilactobacillus salivarius* W24	*Bifidobacterium lactis* W52 *Bifidobacterium lactis* W51 *Bifidobacterium bifidum* W23	*Lactococcus lactis* W19
MS 3	OMNi-BiOTiC^®^ 6, Institut Allergosan, Austria	*Lactobacillus acidophilus* W55 *Ligilactobacillus salivarius* W57 *Lacticaseibacillus casei* W56	*Bifidobacterium animalis* W53	*Enterococcus faecium* W54 *Lactococcus lactis* W58
MS 4	OMNi-BiOTiC^®^ FLORA plus+, Institut Allergosan, Austria	*Lactobacillus crispatus* LBV88 *Lacticaseibacillus rhamnosus* LBV96 *Lactobacillus gasseri* LBV150N *Lactobacillus jensenii* LBV116	*/*	/
MS 5	OMNi-BiOTiC^®^ Activ, Institut Allergosan, Austria	*Lacticaseibacillus casei* W56 *Lactobacillus acidophilus* W37, *Levilactobacillus brevis* W63, *Ligilactobacillus salivarius* W24	*Bifidobacterium lactis* W52, *Bifidobacterium longum* W108, *Bifidobacterium breve* W25, *Bifidobacterium lactis* W51, *Bifidobacterium bifidum* W23	*Lactococcus lactis* W58, *Lactococcus lactis* W19,
MS 6	NutriVital Ultra SB, NutriVital Ply Ltd., Australia	*Lactobacillus acidophilus* La14 *Lactiplantibacillus plantarum* Lp-115	*Bifidobacterium animalis* subsp. *lactis* BI-04	*Saccharomyces cerevisiae* var. *boulardii*
MS 7	(Bio-Kult^®^), Protexin Lopsen Head, UK,	*Lacticaseibacillus casei* PXN 37, *Lactiplantibacillus plantarum* PXN 47, *Lacticaseibacillus rhamnosus* PXN 54, *Lactobacillus acidophilus* PXN 35, *Lactobacillus delbrueckii* subsp. *bulgaricus* PXN 39, *Lactobacillus helveticus* PXN 45, *Ligilactobacillus salivarius* PXN 57	*Bifidobacterium bifidum* PXN 23, *Bifidobacterium breve* PXN 25, *Bifidobacterium longum* PXN 30, *Bifidobacterium infantis* PXN 27	*Bacillus subtilis* PXN 21, *Lactococcus lactis* subsp. *lactis* PXN 63, *Streptococcus thermophilus* PXN 66

^1^ The *Lactobacillus* genus has been recently divided into novel genera [15], therefore the novel genera have been used.

**Table 2:** Single-strain dietary supplements and other beneficial microbes.

Label	Supplement	Strains
SS01	Waya^®^ LGG^®^ forte, Medis GmbH, Austria	*Lacticaseibacillus rhamnosus* LGG
SS02	Yakult^®^, Yakult Honsha Co, Ltd., Yakult Europe, Italy	*Lacticaseibacillus paracasei* Shirota
SS03	BioGaia^®^, TwoPac AB, Sweden	*Limosilactobacillus reuteri* DSM 17938
SS04	German Collection of Microorganisms and Cell Cultures GmbH	*Lactiplantibacillus plantarum* subsp. *plantarum* DSM 2601
SS05	Probactiol^®^ senior, Metagenics Italia S.r.l., Italia	*Bifidobacterium animalis* subsp. *lactis* HN019
SS06	Baby Linbi^®^, Lek Pharmaceutical company d.d., Slovenia	*Bifidobacterium animalis* subsp. *lactis* BB-12
SS07	ProLife^®^ sporogenes, Zeta Farmaceutici, S.p.a., Italy	*Bacillus coagulans* MTCC 5260
SS08	German Collection of Microorganisms and Cell Cultures GmbH	*Propionibacterium freudenreichii subsp. freudenreichii* DSM 20271
SS09	German Collection of Microorganisms and Cell Cultures GmbH	*Acidipropionibacterium acidipropionici* DSM 20272
SS10	Optim PropioniBacter, Laboratoire Optim, Bionoto sprl, Belgium	*Propionibacterium freudenreichii* subsp. *shermanii*
SS11	SB Probiotic, Blooms, Phytologic Pty Ltd., Australia	*Saccharomyces cerevisiae* var. *boulardii*

**Table 3:** Clinical pathogenic isolates and their origin.

Label	Pathogen	Origin
1	*Staphylococcus aureus*	Patient with leg ulcer infection
2		Patient with diabetic ulcer infection
3	*Pseudomonas aeruginosa*	Patient with inguinal infection after cardio intervention
4		Patient with gastrostomy site infection
5	*Enterococcus faecalis*	Patient with infection at central venous catheterization
6		Patient with sternal wound infection
7	*Escherichia coli*	Patient with surgical wound infection and dehiscence
8		Patient with surgical wound infection
9	*Klebsiella pneumoniae*	Patient with sternal wound infection
10		Patient with surgical wound infection
11	*Enterobacter* spp.	Patient with leg ulcer infection
12		Patient with inguinal infection after cardio intervention
13	*Acinetobacter* spp.	Patient with bedsore (pressure ulcer) infection
14	*Bacteroides* spp.	Patient with perianal infection
15		Patient with bedsore (pressure ulcer) infection

**Table 4:** Primer pairs of selected microbial genera or species.

Microorganism	Primer Pairs (5′–3′)		Product Size	Reference
Lactobacilli spp.				
Lactobacilli spp.	LbLMA1-rev	CTC AAA ACT AAA CAA AGT TTC	220 bp	Dubernet et al., 2002 [42]
	R-16-1	CTT GTA CAC ACC GCC CGT C		
*Lacticaseibacillus rhamnosus*	Rham 1	GTC GAA CGA GTT CTG ATT ATT G	158 bp	Sul et al., 2007 [43]
	RhamR	GAA CCA TGC GGT TCT TGG AT		
*Lactobacillus acidophilus*	LacidoF	CAC TTC GGT GAT GAC GTT GG	575 bp	
	LacidoR	CGA TGC AGT TCC TCG GTT AAG C		
*Lacticaseibacillus casei*	PrI	CAG ACT GAA AGT CTG ACG G	200 bp	Walter et al., 2000 [44]
	CasII	GCG ATG CGA ATT TCT TTT TC		
*Limosilactobacillus reuteri*	Lfpr	GCC GCC TAA GGT GGG ACA GAT	350 bp	
	Reu	AAC ACT CAA GGA TTG TCT GA		
*Lactobacillus gasseri*	Lgas-3	AGC GAC CGA GAG AGA GAG A	360 bp	Takahashi et al., 2006 [45]
	Lgas-2	TGC TAT CGC TTC AAG TGC TT		
*Lactiplantibacillus plantarum*	LplanF	CGA GAC AGC AAT TCC TGC ACT CG	176 bp	Gaspar et al., 2019 [46]
	LplanR	CCT CAG AAA CAG TCC GGT TGA C		
Bifidobacteria spp.				
*Bifidobacterium* spp.	Bif164F	GGG TGG TAA TGC CGG ATG	453 bp	Bernhard et al., 2000 [47]
	Bif601R	TAA GCC ATG GAC TTT CAC ACC		
*Bifidobacterium bifidum*	BifF	ATT TGA GCC ACT GTC TGG TG	431 bp	Sul et al., 2007 [43]
	BifR	CAT CCG GGA ACG TCG GGA AA		
*Bifidobacterium longum*	BiflongF	TTC CAG TTG ATC GCA TGG TC	831 bp	
	BiflongR	GGG AAG CCG TAT CTC TAC GA		
*Bifidobacterium animalis*	Bani-tF	TCA CGA CAA GTG GGT TGC CA	178 bp	Sheu et al., 2010 [48]
	Bani-tR	GTT GAT CGG CAG CTT GCC G		
Other bacteria and fungi				
*Lactococcus* spp.	L1	AAC TCT GTT GTT AGA G	570 bp	Deasy et al., 2000 [49]
	L2	ATC TCT AGG AAT AGC AC		
*Propionibacterium* spp.	PB1	AGT GGC GAA GGC GGT TCT CTG GA	865 bp	Rossi et al., 1999 [50]
	PB2	TGG GGT CGA GTT GCA GAC CCC AAT		
*Bacillus coagulans*	BC1-F	ACA GGG CTT TCA GAT ACC CG	990 bp	Majeed et al., 2017 2017 [51]
	BC1-R	CGG GGA TCC GTC CAT CAA AA		
*Bacillus subtilis*	Bsub5F	AAG TCG AGC GGA CAG ATG G	595 bp	Wattiau et al., 2001 [52]
	Bsub5R	CCA GTT TCC AAT GAC CCT CCC C		
*Enterococcus faecium*	EM1F	TTG AGG CAG ACC AGA TTG ACG	658 bp	Cheng et al., 1997 [53]
	EM1R	TAT GAC AGC GAC TCC GAT TCC		
*Saccharomyces cerevisiae*	SC1	AAC GGT GAG AGA TTT CTG TGC	1170 bp	Mitterdorfer et al., 2002 [54]
	SC2	AGC TGG CAG TAT TCC CAC AG		

**Table 5:** Cycling parameters for polymerase chain reaction programs of selected microbes.

PCR Program	Denaturation ^1^	Annealing	Extension	No. of Cycles	Reference/Modified Program
Lactobacilli spp.	30 s at 94 °C	30 s at 55 °C	30 s at 72 °C	20	Dubernet, et al., 2002 [42]
*Lacticaseibacillus casei*, *Limosilalactobacillus reuteri*				30	Walter et al., 2000 [44]
*Lacticaseibacillus rhamnosus*, *Lactobacillus acidophilus*, *Bifidobacterium longum*	30 s at 94 °C	30 s at 63 °C	30 s at 72 °C	30	Sul, et al., 2007 [43]
*Lactobacillus gasseri*	30 s at 94 °C	120 s at 65 °C	120 s at 72 °C	35	Takahashi et al., 2006 [45]
*Lactiplantibacillus plantarum*	15 s at 94 °C	30 s at 60 °C	60 s at 72 °C	40	Gaspar et al., 2019 [46]
*Bifidobacterium* spp.	30 s at 94 °C	60 s at 53 °C	120 s at 72 °C	35	Bernhard et al., 2000 [47]
*Bifidobacterium bifidum*	30 s at 94 °C	45 s at 57 °C	30 s at 72 °C	35	Modified in this study
*Bifidobacterium animalis*	35 s at 94 °C	35 s at 60 °C	40 s at 72 °C	35	Sheu et al., 2010 [48]
*Propionibacterium* spp.	30 s at 94 °C	15 s at 70 °C	60 s at 72 °C	40	Rossi et al., 1999 [50]
*Bacillus coagulans*	30 s at 94 °C	30 s at 60 °C	60 s at 72 °C	30	Majeed et al., 2017 [51]
*Bacillus subtilis*	30 s at 94 °C	120 s at 65 °C	120 s at 72 °C	30	Wattiau et al., 2001 [52]
*Enterococcus faecium*	60 s at 94 °C	60 s at 54 °C	60 s at 72 °C	40	Fijan et al., 2018 [55]
*Lactococcus* spp.	60 s at 94 °C	60 s at 50 °C	60 s at 72 °C	30	Modified in this study
*Saccharomyces cerevisiae*	60 s at 94 °C	60 s at 50 °C	60 s at 72 °C	30	Mitterdorfer et al., 2000 [54]

^1^ Initial denaturation and final extension are 15 min at 95 °C and 7 min at 72 °C respectively for all amplifications.

**Table 6:** Presence of conducted genera-specific and species-specific PCR products of multi-strain probiotics.

Sample	Confirmed Lactobacilli		Confirmed Bifidobacteria		Confirmed Other Bacteria or Fungi	
	Genus-Specific PCR	Species-Specific PCR	Genus-Specific PCR	Species-Specific PCR	Genus-Specific PCR	Species-Specific PCR
MS1	Lactobacilli ^1^ spp.	*L. acidophilus, L. casei*	*Bifidobacterium*	*B. animalis, B. bifidum*	*Lactococcus*	/
MS2	Lactobacilli spp.	*L. acidophilus, L. casei, L. plantarum*	*Bifidobacterium*	*B. animalis, B. bifidum*	*Lactococcus*	/
MS3	Lactobacilli spp.	*L. acidophilus, L. casei*	*Bifidobacterium*	*B. animalis*	*Lactococcus*	*E. faecium*
MS4	Lactobacilli spp.	*L. gasseri, L. rhamnosus*	/	/	/	/
MS5	Lactobacilli spp.	*L. acidophilus, L. casei*	*Bifidobacterium*	*B. animalis, B. bifidum, B. longum*	*Lactococcus*	/
MS6	Lactobacilli spp.	*L. acidophilus, L. plantarum*	*Bifidobacterium*	*B. animalis*	/	*Saccharomyces cerevisiae*
MS7	Lactobacilli spp.	*L. acidophilus, L. casei, L. plantarum, L. rhamnosus*	*Bifidobacterium*	*B. animalis, B. bifidum, B. longum*	*Lactococcus*	*B. subtilis*

^1^ The *Lactobacillus* genus has been recently divided into novel genera [15], therefore the novel genera have been used.

**Table 7:** Presence of conducted genera-specific and species-specific PCR products of single strain microbes.

	Confirmed Genus-Specific PCR	Confirmed Species-Specific PCR
SS01	Lactobacilli spp.^1^	*Lacticaseibacillus rhamnosus*
SS02	Lactobacilli spp.	*Lacticaseibacillus casei*
SS03	Lactobacilli spp.	*Limosilactobacillus reuteri*
SS04	Lactobacilli spp.	*Lactiplantibacillus plantarum*
SS05	*Bifidobacterium* genus	*Bifidobacterium animalis*
SS06	*Bifidobacterium* spp.	*Bifidobacterium animalis*
SS07	(Not conducted)	*Bacillus coagulans*
SS08	*Propionibacterium* spp.	(Not conducted)
SS09	*Propionibacterium* spp.	(Not conducted)
SS10	*Propionibacterium* spp.	(Not conducted)
SS11	(Not conducted)	*Saccharomyces boulardii*

^1^ The *Lactobacillus* genus has been recently divided into novel genera [15], therefore the novel genera have been used.

**Table 8:** Evaluation of the antagonistic effect of various probiotics and other beneficial microbes against clinical skin pathogens using the agar spot assay.

	Evaluation of Zone of Inhibition Using the Agar Spot Assay *														
	Sa1	Sa2	Pa3	Pa4	Ef5	Ef6	Ec7	Ec8	Kp9	Kp10	Ent11	Ent12	Ac13	Bac14	Bac15
MS1	3+	3+	3+	3+	3+	2+	3+	2+	2+	2+	2+	2+	3+	3+	3+
MS2	3+	3+	3+	3+	3+	2+	3+	3+	3+	3+	3+	3+	3+	3+	3+
MS3	3+	3+	3+	3+	3+	3+	3+	3+	3+	3+	3+	2+	3+	3+	3+
MS4	3+	3+	3+	3+	3+	3+	3+	3+	3+	3+	2+	2+	2+	3+	3+
MS5	3+	2+	3+	3+	3+	3+	3+	3+	3+	3+	2+	3+	3+	3+	3+
MS6	3+	3+	3+	3+	3+	3+	3+	3+	3+	3+	2+	3+	3+	3+	3+
MS7	3+	3+	2+	3+	3+	3+	2+	3+	3+	3+	2+	3+	3+	3+	3+
SS01	3+	3+	3+	3+	3+	3+	3+	3+	3+	3+	3+	2+	3+	3+	3+
SS02	3+	3+	3+	3+	3+	3+	3+	3+	3+	2+	2+	2+	3+	3+	3+
SS03	2+	3+	3+	3+	3+	3+	3+	3+	3+	3+	2+	2+	3+	3+	3+
SS04	3+	3+	3+	3+	3+	3+	3+	3+	3+	3+	2+	3+	3+	3+	3+
SS05	2+	2+	2+	2+	2+	2+	2+	3+	3+	2+	3+	3+	2+	2+	3+
SS06	2+	2+	2+	2+	2+	2+	2+	3+	3+	3+	3+	3+	2+	3+	2+
SS07	+	2+	2+	2+	+	+	+	+	+	+	+	+	+	+	+
SS08	+	+	+	+	+	+	+	+	+	+	+	+	+	+	+
SS09	+	+	+	+	+	+	+	+	+	+	+	+	+	+	+
SS10	+	+	+	+	+	+	+	+	+	+	+	+	+	+	+
SS11	2+	2+	3+	3+	2+	3+	3+	2+	2+	2+	+	+	2+	2+	2+

* More than 20 mm was considered strong inhibition (3+), between 11 and 20 mm was considered intermediate inhibition (2+), and less than 10 mm was considered low inhibition (+). The diameter of the colony is included. If no zone of inhibition was detected the result was reported as <6 mm.

**Table 9:** Evaluation of the antagonistic effect of various probiotics and other beneficial microbes against clinical skin pathogens using the co-culturing assay.

	Evaluation of Log Step Reduction Using the Co-Culturing Assay *														
	Sa1	Sa2	Pa3	Pa4	Ef5	Ef6	Ec7	Ec8	Kp9	Kp10	Ent11	Ent12	Ac13	Bac14	Bac15
MS1	2+	2+	3+	3+	2+	2+	3+	2+	+	2+	3+	3+	3+	3+	3+
MS2	2+	2+	3+	3+	2+	2+	2+	2+	+	2+	3+	3+	3+	3+	2+
MS3	2+	2+	2+	3+	2+	2+	3+	3+	+	3+	3+	3+	3+	3+	2+
MS4	2+	2+	3+	3+	2+	2+	3+	3+	3+	3+	2+	3+	3+	3+	3+
MS5	2+	2+	3+	3+	+	2+	3+	3+	3+	2+	3+	3+	3+	2+	3+
MS6	2+	2+	3+	2+	+	2+	2+	+	+	2+	2+	3+	3+	2+	+
MS7	2+	2+	3+	3+	2+	2+	3+	3+	3+	3+	3+	3+	3+	2+	2+
SS01	2+	+	3+	3+	+	+	2+	2+	2+	+	2+	3+	+	2+	+
SS02	2+	2+	3+	3+	2+	+	2+	2+	2+	+	3+	3+	2+	2+	+
SS03	2+	2+	3+	3+	2+	2+	2+	3+	+	2+	3+	3+	2+	+	+
SS04	3+	3+	3+	2+	2+	2+	3+	3+	3+	2+	3+	3+	2+	3+	2+
SS05	2+	2+	3+	2+	2+	2+	3+	3+	+	3+	3+	3+	2+	3+	2+
SS06	3+	3+	3+	3+	+	2+	2+	3+	2+	3+	3+	3+	2+	+	+
SS07	2+	+	3+	3+	2+	+	2+	2+	2+	2+	2+	2+	2+	+	+
SS08	+	+	+	+	+	+	+	+	+	+	+	+	2+	2+	+
SS09	+	+	+	+	+	+	+	+	+	+	+	+	+	+	+
SS10	2+	+	2+	+	+	+	+	2+	2+	2+	2+	2+	2+	+	3+
SS11	2+	2+	+	+	+	+	2+	+	+	+	+	2+	+	+	+

* a log step reduction of more than 6 was considered strong inhibition (3+), between 3 and 6 was considered intermediate inhibition (2+) and less than 3 was considered low inhibition (+).

**Table 10:** Evaluation of the antagonistic effect of various cell-free supernatants of probiotics and other beneficial microbes against clinical skin pathogens using the agar well diffusion assay.

	Evaluation of Zone of Inhibition Using the Agar Well Diffusion Assay *														
	Sa1	Sa2	Pa3	Pa4	Ef5	Ef6	Ec7	Ec8	Kp9	Kp10	Ent11	Ent12	Ac13	Bac14	Bac15
MS1	2+	2+	2+	2+	2+	2+	2+	2+	2+	2+	2+	+	2+	2+	+
MS2	2+	2+	+	+	+	+	2+	2+	2+	2+	2+	+	+	–	+
MS3	2+	+	+	2+	2+	+	2+	2+	+	2+	2+	+	+	2+	+
MS4	2+	+	+	2+	2+	–	2+	2+	+	2+	2+	2+	+	+	2+
MS5	2+	+	–	2+	+	–	2+	2+	+	2+	+	2+	+	+	2+
MS6	+	+	2+	–	+	+	+	3+	+	+	2+	2+	+	+	2+
MS7	2+	2+		2+	–	2+	2+	+	2+	+	+	+	+	+	+
SS01	2+	2+	2+	+	–	–	2+	3+	+	+	+	–	+	–	+
SS02	2+	+	+	2+	+	+	2+	2+	+	2+	+	2+	+	–	–
SS03	–	2+	+	+	–	–	2+	2+	+	–	2+	2+	+	–	+
SS04	2+	+	–	+	+	–	+	2+	+	+	2+	+	+	–	–
SS05	2+	2+	2+	+	–	–	2+	2+	2+	2+	+		+	–	–
SS06	+	–		+	+	+	2+	2+	2+	2+	2+	2+	2+	–	–
SS07	–	+	2+	2+	–	–	–	–	–	–	+	–	+	2+	–
SS08	+	+	–	–	–	–	+	–	–	–	2+	–	–	–	–
SS09	+	2+	–	–	–	–	+	–	–	–	–	–	–	–	–
SS10	+	+	2+	+	–	2+	+	+	+	+	–	+	–	+	–
SS11	+	–	2+	2+	+	–	–	2+	–	–	+	–	+	+	–

* Cell-free supernatant after filtration; more than 20 mm was considered strong inhibition (3+), between 11 and 20 mm was considered intermediate inhibition (2+), and less than 10 mm was considered low inhibition (+). The diameter of the colony is included. If no zone of inhibition was detected the result was reported as <6 mm (–).

**Table 11:** Evaluation of the antagonistic effect of various neutralized cell-free supernatants of probiotics and other beneficial microbes against clinical skin pathogens using the agar well diffusion assay.

	Evaluation of Zone of Inhibition Using the Agar Well Diffusion Assay *														
	Sa1	Sa2	Pa3	Pa4	Ef5	Ef6	Ec7	Ec8	Kp9	Kp10	Ent11	Ent12	Ac13	Bac14	Bac15
MS1	–	+	2+	–	–	–	–	2+	–	–	–	–	–	–	–
MS2	–	+	+	+	+	–	2+	2+	–	2+	–	–	+	2+	+
MS3	–	+	+	+	2+	+	2+	+	2+	2+	–	–	+	–	+
MS4	–	+	–	–	+	+	–	2+	–	2+	2+	2+	–	–	2+
MS5	–	–	–	–	–	–	–	–	–	2+	–	+	–	–	2+
MS6	+	–	–	–	–	–	–	–	–	–	–	–	+	–	2+
MS7	+	2+	–	–	2+	2+	2+	–	–	–	+	2+	–	–	–
SS01	–	2+	+	2+	–	–	2+	2+	–	–	2+	–	–	–	–
SS02	–	2+	–	–	–	–	2+	2+	–	–	2+	–	–	3+	–
SS03	–	2+	–	2+	–	–	2+	2+	–	–	2+	2+	–	–	–
SS04	+	2+	–	–	–	–	+	2+	–	2+	2+	–	+	–	–
SS05	+	+	–	–	–	–	–	2+	–	–	2+	–	+	2+	–
SS06	+	–	–	–	2+	–	2+	+	2+	2+	+	+	+	–	2+
SS07	+	+	–	–	–	–	–	–	–	–	–	–	+	–	–
SS08	2+	+	–	–	–	–	–	–	–	–	2+	–	–	–	–
SS09	+	2+	–	–	–	–	+	+	–	–	2+	–	–	–	–
SS10	–	+	–	–	–	–	+	+	2+	–	–	–	–	–	–
SS11	+	–	2+	–	–	–	–	–	–	–	–	–	+	–	–

* Neutralized cell-free supernatant after filtration with pH = 7 by addition of NaOH. More than 20 mm was considered strong inhibition (3+), between 11 and 20 mm was considered intermediate inhibition (2+), and less than 10 mm was considered low inhibition (+). The diameter of the colony is included. If no zone of inhibition was detected, the result was reported as <6 mm.

## Data Availability

No additional data is available.

## Supplementary Materials

The following supporting information can be downloaded at: https://www.mdpi.com/article/10.3390/microorganisms10122518/s1, (Tables S1–S4). S1: Results of the antagonistic effect of various probiotics and other beneficial microbes against clinical skin pathogens using the agar spot assay. S2: Results of the antagonistic effect of various probiotics and other beneficial microbes against clinical skin pathogens using the co-culturing assay. S3: Results of the antagonistic effect of various cell-free supernatants of probiotics and other beneficial microbes against clinical skin pathogens using the agar well diffusion assay. S4: Results of the antagonistic effect of various neutralised cell-free supernatants of probiotics and other beneficial microbes against clinical skin pathogens using the agar well diffusion assay.
