# Supplementary material for: The Antimicrobial Effect of Various Single-Strain and Multi-Strain Probiotics, Dietary Supplements or Other Beneficial Microbes against Common Clinical Wound Pathogens
Source: Microorganisms. 2022 Dec 19;10(12):2518. doi: 10.3390/microorganisms10122518 (PMC9781324; doi:10.3390/microorganisms10122518)
Supplement: Supplementary file 1 [file microorganisms-10-02518-s001.zip › microorganisms-2028387-supplementary.pdf]

Table S1: Results of the antagonistic effect of various probiotics and other beneficial microbes against clinical skin pathogens using the agar spot assay

	Mean zone of inhibition (standard deviation) mm*															AV_{ZOIpro}
	Sa1	Sa2	Pa3	Pa4	Ef5	Ef6	Ec7	Ec8	Kp9	Kp10	Ent11	Ent12	Ac13	Bac14	Bac15	
MS1	27.33 (1.15)	26.5 (1)	26 (0.82)	25.5 (1)	22.5 (4.12)	16.67 (1.15)	27.33 (1.15)	19.33 (1.15)	17.33 (1.15)	14.67 (2.31)	14.33 (0.58)	16.67 (1.15)	36.67 (1.15)	22 (2.31)	27.33 (2.31)	**22.68**
MS2	33.33 (1.15)	24.5 (1)	22.67 (1.15)	25 (1.41)	22.67 (3.06)	19.33 (1.15)	26.67 (1.15)	25.67 (0.58)	21.33 (1.15)	26.67 (3.06)	20.67 (2.31)	24.67 (4.16)	30.13 (0.25)	28.5 (0.71)	27.33 (1.15)	**25.28**
MS3	29.5 (2.52)	23.75 (1.26)	23.33 (1.15)	29.5 (2.52)	24.67 (2.31)	27.33 (1.15)	27.0 (1.41)	30.0 (3.46)	28.5 (3.79)	23.33 (3.06)	24.67 (1.15)	19.33 (3.06)	35.33 (3.06)	29.33 (2.31)	36.5 (0.71)	**27.47**
MS4	25.33 (1.15)	20.67 (1.15)	24.0 (2)	28.0 (3.46)	24.25 (0.5)	22.33 (0.58)	22.67 (5.03)	26.0 (2)	23.33 (1.15)	24.0 (2)	14.67 (2.31)	19.33 (1.15)	18.67 (1.15)	22.5 (0.71)	30 (1.63)	**23.05**
MS5	29.5 (1)	18.5 (1)	25.33 (1.15)	32.25 (0.5)	21.0 (2)	22.67 (3.06)	24.67 (2.31)	25.33 (1.15)	25.33 (1.15)	28.0 (1)	14.67 (1.15)	22.0 (4)	36.67 (1.15)	28.5 (1.91)	26.67 (1.15)	**25.41**
MS6	27.33 (1.15)	24.5 (1)	25.0 (1.41)	25.0 (2)	35.00 (1.41)	26.25 (0.5)	29.0 (1.41)	29.0 (1.41)	30.0 (2.83)	20.67 (3.06)	15.67 (0.58)	24.67 (4.16)	41.0 (1.41)	26.5 (0.71)	36.0 (1)	**27.71**
MS7	20.5 (1)	23.0 (2)	16.67 (2.31)	26.67 (1.15)	22.0 (5.66)	26.67 (2.31)	17.5 (0.71)	26.67 (1.15)	24.0 (2.83)	23.33 (1.15)	16.67 (1.15)	25.33 (1.15)	41.5 (0.71)	25.33 (1.15)	31.67 (0.58)	**24.50**
SS01	32.0 (2.83)	20.5 (2.52)	26.25 (0.5)	29.33 (1.15)	25.5 (3)	26.33 (0.58)	27.33 (1.15)	25.0 (1.41)	25.67 (0.58)	27.33 (1.15)	20.67 (1.15)	18.67 (3.06)	35.0 (4.24)	26.13 (0.25)	29.33 (1.15)	**26.34**
SS02	23.0 (7.07)	20.67 (1.15)	26.67 (1.15)	24.5 (3)	24.67 (1.15)	24.67 (1.15)	24.5 (1.91)	27.0 (1.15)	21.0 (2.58)	18.67 (1.15)	14.5 (1.91)	13.33 (1.15)	34.0 (2)	27.0 (2)	24.67 (1.15)	**23.26**
SS03	19.33 (1.15)	21.5 (1)	27.5 (1)	27.0 (2)	29.0 (1.41)	24.33 (0.58)	28.67 (1.15)	24.0 (1.63)	29.67 (0.58)	24.0 (2)	12.67 (1.15)	13.33 (1.15)	39.0 (1.41)	25.5 (1)	25.33 (1.15)	**24.72**
SS04	29.5 (1)	24.5 (1)	20.5 (3.79)	29.5 (1.91)	23.5 (1)	26.0 (2)	27.33 (2.31)	29.0 (1.41)	22.5 (0.71)	22.67 (1.15)	17.67 (0.58)	24.0 (2.83)	42.5 (0.71)	30.0 (4)	32.67 (4.16)	**26.79**
SS05	16.25 (0.5)	18.25 (0.5)	16.25 (0.5)	15.25 (0.96)	13.8 (0.45)	13.25 (0.96)	11.5 (1)	21.25 (0.96)	20.25 (0.5)	18.2 (0.45)	21.0 (2.58)	20.67 (3.06)	18.2 (0.45)	18.2 (0.45)	20.5 (1)	**17.52**
SS06	14.75 (0.96)	16.25 (0.5)	17.8 (0.45)	17.25 (0.96)	11.75 (1.26)	14.25 (2.06)	16.0 (1.63)	22.0 (1.63)	21.8 (0.45)	23.5 (1)	22.0 (1.63)	22.0 (2)	20.75 (0.96)	20.25 (0.5)	18.2 (0.45)	**18.57**
SS07	8.25 (0.5)	12.0 (1.63)	10.5 (1)	10.33 (0.58)	8.25 (0.5)	8.2 (0.45)	8.6 (0.89)	8.2 (0.45)	7.67 (0.58)	8.5 (0.71)	7.33 (0.58)	7.67 (0.58)	7.33 (0.58)	7.67 (0.58)	7.67 (0.58)	**8.54**
SS08	7.2 (0.45)	8.75 (0.5)	7 (1)	7.5 (1)	6.25 (0.5)	6.2 (0.45)	6.75 (0.5)	6.75 (0.5)	7.75 (0.5)	6.5 (0.58)	7.75 (0.5)	7.5 (0.58)	7.75 (0.5)	7.25 (0.5)	7.2 (0.45)	**7.21**
SS09	7.4 (0.89)	6.63 (0.48)	6.33 (0.58)	6.5 (1)	6.25 (0.5)	6.25 (0.5)	6.88 (0.25)	6.88 (0.25)	7.13 (0.63)	6.63 (0.48)	7.8 (0.45)	7.75 (0.5)	6.8 (0.45)	7.4 (0.55)	7.33 (0.52)	**6.93**
SS10	7.25 (0.5)	6.38 (0.48)	6.25 (0.5)	6.67 (1.15)	6.75 (0.5)	6.75 (0.5)	6.75 (0.5)	6.75 (0.5)	7.25 (0.5)	6.75 (0.5)	8.6 (0.89)	8.5 (1)	7.1 (0.55)	8.4 (0.89)	8.33 (0.82)	**7.23**
SS11	19.5 (1)	20.5 (1)	24.25 (0.5)	27.6 (0.89)	18.5 (1)	24.25 (0.5)	24.13 (0.25)	16.5 (1)	16.0 (1.63)	13.5 (1)	8.5 (1)	8.25 (0.5)	10.5 (1)	11.25 (1.5)	11.5 (1.91)	**16.98**
AV_{ZOIpa}	**20.58**	**18.29**	**19.19**	**21.64**	**19.05**	**19.12**	**19.76**	**20.94**	**19.95**	**18.96**	**15.03**	**16.88**	**25.43**	**20.57**	**22.41**	

*More than 20 mm was considered strong inhibition (3+), between 11 and 20 mm was considered intermediate inhibition (2+) and less than 10 mm was considered low inhibition (+). The diameter of the colony is included. If no zone of inhibition was detected the result was reported as < 6 mm.

AV_{ZOIpa} means average zone of inhibition of all probiotics against the individual pathogen.
AV_{ZOIpro} means average zone of inhibition of individual probiotic against all pathogens.

Table S2: Results of the antagonistic effect of various probiotics and other beneficial microbes against clinical skin pathogens using the co-culturing assay

	Log step reduction (Percentage of reduction) *															AV$_{ZOIpro}$
	Sa1	Sa2	Pa3	Pa4	Ef5	Ef6	Ec7	Ec8	Kp9	Kp10	Ent11	Ent12	Ac13	Bac14	Bac15	
MS1	4.37 (99.9958%)	3.58 (99.9734%)	7.66 (100%)	7.12 (99.9999%)	3.2 (99.9367%)	4.09 (99.9918%)	6.8 (99.99998%)	4.43 (99.9963%)	1.8 (98.42487%)	5.91 (99.9999%)	7.7 (100%)	7.74 (100%)	6.62 (99.99998%)	7.71 (100%)	7.47 (100%)	5.75
MS2	4.87 (99.9986%)	3.89 (99.9871%)	7.66 (100%)	7.21 (99.99999%)	3.35 (99.9558%)	4.21 (99.9938%)	4.58 (99.9974%)	4.98 (99.999%)	2.66 (99.78187%)	3.86 (99.9863%)	7.65 (100%)	7.68 (100%)	6.32 (99.99995%)	7.71 (100%)	4.49 (99.9967%)	5.41
MS3	4.37 (99.9958%)	4.44 (99.9964%)	3.67 (99.9786%)	2.13 (99.26129%)	3.43 (99.9625%)	4.44 (99.9964%)	6.85 (99.99999%)	7.05 (100%)	2.43 (99.62591%)	7.4 (100%)	6.31 (99.99995%)	7.79 (100%)	6.68 (99.99998%)	7.54 (100%)	3.16 (99.9304%)	**5.18**
MS4	5.94 (99.9999%)	5.83 (99.9999%)	7.66 (100%)	7.08 (99.99999%)	4.18 (99.9933%)	5.12 (99.9992%)	6.86 (99.99999%)	6.92 (100%)	6.89 (99.99999%)	6.23 (99.9999%)	5.31 (99.9995%)	7.74 (100%)	6.57 (99.99997%)	6.83 (99.99999%)	7.17 (100%)	**6.42**
MS5	5.86 (99.9999%)	4.66 (99.9978%)	7.66 (100%)	6.5 (99.99997%)	2.16 (99.3032%)	5.29 (99.9995%)	6.69 (99.99998%)	6.88 (100%)	6.45 (99.99996%)	3.29 (99.9486%)	7.6 (100%)	7.49 (100%)	9.04 (100%)	5.2 (99.99936%)	7.01 (100%)	**6.12**
MS6	3.86 (99.9861%)	3.64 (99.9771%)	7.66 (100%)	3.61 (99.97558%)	2.23 (99.4167%)	3.93 (99.9882%)	5.59 (99.99974%)	1.32 (95.1642%)	2.3 (99.50052%)	3.22 (99.9394%)	5.65 (99.99978%)	6.13 (99.99993%)	7.91 (100%)	4.04 (99.99093%)	1.37 (95.7216%)	**4.16**
MS7	4.66 (99.9978%)	3.95 (99.9889%)	7.66 (100%)	6.94 (99.99999%)	3.51 (99.9692%)	4.78 (99.9983%)	6.81 (99.99998%)	6.98 (100%)	7.94 (100%)	6.46 (100%)	7.76 (100%)	7.71 (100%)	8.68 (100%)	5.94 (99.99988%)	4.9 (99.9987%)	**6.31**
SS01	3.5 (99.9685%)	2.48 (99.6707%)	6.23 (99.9999%)	7.21 (100%)	1.89 (98.7011%)	1.44 (96.3556%)	4.29 (99.99491%)	4.12 (99.9924%)	3.61 (99.97533%)	2.6 (99.7494%)	5.9 (99.99988%)	7.08 (99.99999%)	1.98 (98.9494%)	4.6 (99.99747%)	1.76 (98.2615%)	**3.91**
SS02	3.76 (99.9827%)	3.18 (99.9346%)	6.32 (100%)	6.95 (99.99999%)	3.24 (99.9419%)	2.62 (99.7611%)	4.48 (99.99671%)	3.94 (99.9884%)	4.92 (99.99881%)	2.03 (99.0694%)	6.97 (99.99999%)	7.63 (100%)	3.92 (99.98795%)	4.48 (99.99672%)	1.54 (97.1341%)	**4.40**
SS03	4.77 (99.9983%)	4.45 (99.9964%)	6.18 (99.9999%)	6.87 (99.99999%)	3.68 (99.9792%)	4.97 (99.9989%)	5.49 (99.99968%)	6.87 (100%)	1.27 (94.57254%)	4.76 (99.9983%)	6.07 (99.99992%)	6.24 (99.99994%)	5.98 (99.9998%)	0.59 (74%)	2.74 (99.8183%)	**4.73**
SS04	6.41 (99.99996%)	6.09 (99.9999%)	6.4 (100%)	4.39 (99.99588%)	4.73 (99.9981%)	3.35 (99.9555%)	6.16 (99.99993%)	7.08 (100%)	6.49 (99.99997%)	5.59 (99.9997%)	7.32 (100%)	6.73 (99.99998%)	5.52 (99.9997%)	7.35 (100%)	4.82 (99.9985%)	**5.90**
SS05	4.79 (99.9984%)	4.36 (99.9956%)	6.36 (100%)	5.93 (99.99988%)	3.53 (99.9704%)	4.34 (99.9955%)	6.5 (99.99997%)	6.98 (100%)	1.36 (95.66062%)	6.54 (100%)	7.79 (100%)	6.05 (99.99991%)	5.52 (99.9997%)	7.71 (100%)	5.93 (99.9999%)	**5.58**
SS06	6.82 (99.9999848%)	6.07 (99.9999%)	7.17 (100%)	6.91 (99.99999%)	2.08 (99.1667%)	4.69 (99.998%)	5.23 (99.99942%)	6.82 (100%)	5.88 (99.99987%)	6.08 (99.9999%)	7.38 (100%)	7.43 (100%)	4.98 (99.99895%)	2.61 (99.7529%)	1.37 (95.7555%)	**5.43**
SS07	5.1 (99.9992%)	2.82 (99.849%)	7.87 (100%)	6.85 (99.99999%)	3.31 (99.9505%)	2.29 (99.4889%)	4.56 (99.99727%)	4.85 (99.9986%)	5.81 (99.99985%)	5.11 (99.9992%)	5.3 (99.9995%)	5.44 (99.99964%)	5.04 (99.9991%)	1.4 (96%)	1.07 (91.511%)	**4.45**
SS08	2.22 (99.3922%)	1.43 (96.2764%)	0.81 (84.64%)	1.1 (92.0202%)	0.87 (86.5789%)	1.06 (91.2258%)	1.89 (98.7%)	1.91 (98.7812%)	1.77 (98.29545%)	1.55 (97.1833%)	1.94 (98.84932%)	2.01 (99.02056%)	3.59 (99.9741%)	3.98 (99.98942%)	0.31 (51.1036%)	**1.76**
SS09	1.95 (98.8816%)	1.79 (98.3927%)	2.26 (99.4533%)	2.11 (99.22828%)	0.66 (78.0263%)	1.08 (91.6903%)	1.6 (97.4625%)	1.68 (97.8918%)	2.29 (99.48864%)	1.63 (97.6667%)	2.05 (99.11233%)	2.23 (99.40935%)	1.78 (98.3494%)	1.75 (98.22394%)	0.67 (78.7776%)	**1.70**
SS10	3.6 (99.9748%)	2.24 (99.4273%)	4.78 (99.9983%)	2.15 (99.29293%)	0.19 (35.5263%)	1.25 (94.3484%)	1.96 (98.90625%)	3.07 (99.9158%)	5.69 (99.9998%)	3.46 (99.9654%)	4.99 (99.99897%)	4.11 (99.99224%)	5.5 (99.99968%)	0.54 (71.4%)	4.29 (99.9949%)	**3.19**
SS11	4.44 (99.9964%)	3.22 (99.94%)	2.76 (99.8277%)	1.55 (97.16117%)	1.72 (98.0917%)	1.26 (94.5152%)	4.43 (99.99626%)	1.31 (95.0896%)	0.47 (66.11399%)	1.33 (95.3613%)	2.77 (99.82921%)	5.04 (99.99908%)	1.59 (97.40361%)	1.4 (95.98456%)	0.48 (66.7233%)	**2.25**
AV$_{ZOIpa}$	**4.52**	**3.78**	**5.93**	**5.15**	**2.66**	**3.35**	**5.04**	**4.84**	**3.89**	**4.28**	**5.91**	**6.24**	**5.40**	**4.52**	**3.48**	

*a log step reduction of more than 6 was considered strong inhibition, between 3 and 6 was considered intermediate inhibition and less than 3 was considered low inhibition. AV$_{ZOIpa}$ means average log step reduction of all probiotics against the individual pathogen. AV$_{ZOIpro}$ means average log step reduction of individual probiotic against all pathogens.

Table S3: Results of the antagonistic effect of various cell-free supernatants of probiotics and other beneficial microbes against clinical skin pathogens using the agar well diffusion assay

	Mean zone of inhibition (standard deviation) mm*															AV_{ZOIpro}
	Sa1	Sa2	Pa3	Pa4	Ef5	Ef6	Ec7	Ec8	Kp9	Kp10	Ent11	Ent12	Ac13	Bac14	Bac15	
MS1	13.67 (5.96)	10.33 (2.06)	12.5 (1)	12 (3.27)	12.67 (4.13)	10.33 (3.67)	12.6 (3.27)	10 (1.85)	10 (1.41)	14.25 (1.28)	10.75 (3.2)	9.43 (2.51)	12 (3.21)	15 (6.26)	7.25 (0.5)	**11.52**
MS2	14 (6.03)	13.82 (2.6)	7.25 (0.5)	7.22 (0.83)	9.2 (3.03)	6.83 (0.75)	10.44 (2.6)	12 (4.73)	11 (3.95)	12.86 (4.88)	15 (5.76)	9.33 (2.73)	9 (2.65)	< 6	7 (0)	**10.06**
MS3	10.83 (4.04)	7.27 (0.65)	7.38 (0.92)	16.83 (4.55)	18.8 (8.07)	8 (1.79)	11.43 (3.78)	14.67 (6.77)	6.5 (0.58)	11.71 (4.07)	17.78 (8.39)	9.67 (2.94)	9.71 (3.45)	20.33 (10.52)	6.5 (0.58)	**11.83**
MS4	11.83 (4.47)	9.33 (1.3)	9.71 (3.55)	10 (2.31)	14 (5.57)	< 6	12.22 (3.67)	13.71 (0.76)	9.43 (2.99)	12 (2.62)	14.67 (2)	13.11 (2.03)	9.75 (3.2)	6.5 (0.58)	17 (1.15)	**11.28**
MS5	11.83 (5.01)	9.83 (3.01)	< 6	12.8 (3.79)	9.67 (3.2)	< 6	11.5 (2.56)	10.33 (3.44)	9.67 (2.94)	15.5 (4.87)	9.5 (2.56)	10 (3.02)	9.22 (1.86)	6.5 (0.58)	19 (1.15)	**10.49**
MS6	8.55 (3.8)	8.67 (2.42)	16 (1.63)	< 6	7.33 (1.03)	6.67 (0.52)	7 (0)	21 (3.38)	7 (1.15)	6.33 (0.58)	18.67 (9.85)	18 (9.38)	8 (1.1)	6.5 (0.58)	18.5 (1.91)	**10.95**
MS7	14.6 (5.97)	10.5 (1.24)	8.5 (2.78)	13.67 (2.06)	< 6	15.2 (9.34)	13.09 (3.51)	7.33 (1.03)	11.33 (4.32)	7.25 (0.46)	8.5 (1.6)	8.67 (2.07)	7.67 (1.51)	6.5 (0.58)	8 (0)	**9.79**
SS01	11.08 (5.2)	10 (4.37)	17 (2.58)	8.8 (1.69)	< 6	< 6	16.6 (2.84)	21.71 (4.96)	6.33 (0.58)	7 (0)	8.67 (2.07)	< 6	7 (1)	< 6	7 (0)	**9.68**
SS02	13.8 (1.99)	8.58 (2.81)	7.75 (0.5)	10.2 (2.39)	8 (1.79)	7.17 (0.98)	13.67 (4.8)	12 (4.87)	6.8 (0.84)	11.25 (4.1)	8.67 (1)	10.25 (1.28)	7.67 (0.52)	< 6	< 6	**9.19**
SS03	< 6	14 (4.69)	9.5 (3.08)	6.78 (0.44)	< 6	< 6	19.09 (2.07)	17 (8.56)	6.33 (0.58)	< 6	12.67 (5.16)	15 (6.22)	6.5 (0.58)	< 6	7 (0)	**9.59**
SS04	10 (2.49)	6.89 (0.33)	< 6	9 (1.7)	7 (0)	< 6	6.5 (0.58)	10 (2.83)	6.5 (0.58)	6.67 (0.52)	10 (2.83)	8.67 (2.07)	8.75 (2.12)	< 6	< 6	**7.60**
SS05	11.83 (4.86)	11.56 (2.6)	11.2 (4.82)	6.8 (0.42)	< 6	< 6	14.4 (3.24)	10 (3.1)	10.29 (3.15)	10.86 (3.63)	8.33 (2.34)	< 6	7.6 (1.67)	< 6	< 6	**8.86**
SS06	8.18 (3.16)	< 6	9 (1.15)	9 (2.45)	6.67 (0.52)	6.67 (1.03)	13 (5.23)	14 (5.95)	11.75 (4.65)	11 (1.15)	10 (1.63)	11.6 (1.67)	11.5 (1.91)	< 6	< 6	**9.36**
SS07	< 6	7.5 (1)	14.5 (6.61)	10.17 (4.78)	< 6	< 6	< 6	< 6	< 6	< 6	9.5 (3.42)	< 6	6.6 (0.55)	15 (2.58)	< 6	**7.82**
SS08	7.75 (1.98)	8 (1.63)	< 6	< 6	< 6	< 6	7.33 (1.03)	< 6	< 6	< 6	16.5 (1.91)	< 6	< 6	< 6	< 6	**7.04**
SS09	7.71 (2.43)	11.6 (3.29)	< 6	< 6	< 6	< 6	7.33 (1.03)	< 6	< 6	< 6	< 6	< 6	< 6	< 6	< 6	**6.58**
SS10	9.38 (1.92)	8 (1.95)	10 (1.63)	9.2 (2.28)	< 6	14 (6.32)	9.1 (1.2)	7.33 (0.5)	8 (2)	7.43 (1.4)	< 6	7.5 (0.53)	< 6	8 (1.63)	< 6	**8.13**
SS11	6.33 (0.82)	< 6	17 (1.15)	13 (6.03)	6.67 (0.52)	< 6	< 6	12.33 (4.97)	< 6	< 6	8.67 (2.07)	< 6	8.17 (2.04)	< 6	< 6	**8.01**
AV_{ZOIpa}	**10.19**	**9.33**	**10.07**	**9.64**	**8.22**	**7.49**	**10.96**	**11.75**	**8.05**	**9.12**	**11.10**	**9.29**	**8.17**	**8.02**	**8.40**	

*Cell-free supernatant after filtration; More than 20 mm was considered strong inhibition (3+), between 11 and 20 mm was considered intermediate inhibition (2+) and less than 10 mm was considered low inhibition (+). The diameter of the colony is included. If no zone of inhibition was detected the result was reported as < 6 mm.

AV_{ZOIpa} means average zone of inhibition of all probiotics against the individual pathogen.
AV_{ZOIpro} means average zone of inhibition of individual probiotic against all pathogens.

Table S4: Results of the antagonistic effect of various neutralised cell-free supernatants of probiotics and other beneficial microbes against clinical skin pathogens using the agar well diffusion assay

	Mean zone of inhibition (standard deviation) mm*															AV_{ZOIpro}
	Sa1	Sa2	Pa3	Pa4	Ef5	Ef6	Ec7	Ec8	Kp9	Kp10	Ent11	Ent12	Ac13	Bac14	Bac15	
MS1	< 6	7.14 (1.07)	17 (1.15)	< 6	< 6	< 6	< 6	12 (4.9)	< 6	< 6	< 6	< 6	7 (1)	< 6	< 6	**7.28**
MS2	< 6	8.86 (2.79)	7.67 (0.58)	6.6 (0.55)	9 (2.45)	< 6	15.5 (1)	13 (1.15)	< 6	12.67 (1.15)	< 6	< 6	7.14 (0.69)	1< 6	7.75 (0.5)	**8.95**
MS3	< 6	8.29 (2.14)	9.14 (2.91)	8.4 (1.67)	10.67 (4.13)	6.25 (0.5)	12 (1.63)	9 (1.15)	12.67 (1.15)	14.67 (2.31)	< 6	< 6	7.33 (0.52)	< 6	8 (0)	**8.69**
MS4	< 6	7.43 (1.51)	< 6	< 6	6.67 (0.52)	6.33 (0.58)	< 6	14.5 (3)	< 6	16 (2)	16 (1.63)	15 (3.46)	< 6	< 6	15.5 (1.91)	**9.30**
MS5	< 6	< 6	< 6	< 6	< 6	< 6	< 6	< 6	< 6	14 (3.65)	< 6	8.5 (3)	< 6	< 6	18.67 (6.11)	**7.54**
MS6	8.55 (3.8)	< 6	< 6	< 6	< 6	< 6	< 6	< 6	< 6	< 6	< 6	< 6	7 (0)	< 6	19.33 (1.15)	**7.13**
MS7	6.25 (0.5)	11 (3.83)	< 6	< 6	18 (11.05)	13.2 (6.57)	13 (3.46)	< 6	< 6	< 6	7 (0)	10.5 (5.26)	< 6	< 6	< 6	**8.46**
SS01	< 6	11.2 (3.35)	7.75 (0.5)	14.8 (7.29)	< 6	< 6	16 (1.79)	14 (6.2)	< 6	< 6	12 (5.48)	< 6	< 6	< 6	< 6	**8.65**
SS02	< 6	10.67 (4.16)	< 6	< 6	< 6	< 6	11.6 (5.18)	12.33 (4.97)	< 6	< 6	10.8 (4.38)	< 6	< 6	24 (2)	< 6	**8.63**
SS03	< 6	13.5 (5.26)	< 6	18.8 (7.29)	< 6	< 6	15.78 (2.11)	12 (4.73)	< 6	< 6	14 (2)	13.5 (6.61)	< 6	< 6	< 6	**9.44**
SS04	8.57 (4.43)	10.5 (3.42)	< 6	< 6	< 6	< 6	6.6 (0.55)	12 (0)	< 6	11 (1.15)	11 (1.15)	< 6	9.75 (1.67)	< 6	< 6	**7.83**
SS05	7.09 (1.87)	8.4 (1.67)	< 6	< 6	< 6	< 6	< 6	12.33 (4.97)	< 6	< 6	13 (1.15)	< 6	8 (0)	17 (1.15)	< 6	**7.99**
SS06	8.17 (1.8)	< 6	< 6	< 6	11.33 (4.32)	< 6	12.57 (4.58)	9.33 (2.73)	11 (3.95)	13.75 (2.25)	7.2 (1.1)	8.4 (2.19)	7.67 (1.51)	< 6	11.5 (6.4)	**8.73**
SS07	8.5 (2.78)	9.2 (2.28)	< 6	< 6	< 6	< 6	< 6	< 6	< 6	< 6	< 6	< 6	8 (0)	< 6	< 6	**6.51**
SS08	10.86 (6.09)	9.6 (2.19)	< 6	< 6	< 6	< 6	< 6	< 6	< 6	< 6	17 (3.46)	< 6	< 6	< 6	< 6	**7.30**
SS09	6.5 (0.53)	10.5 (3.42)	< 6	< 6	< 6	< 6	7.2 (1.1)	8 (0)	< 6	< 6	16 (1.41)	< 6	< 6	< 6	< 6	**7.55**
SS10	< 6	9.6 (2.19)	< 6	< 6	< 6	< 6	7.13 (0.83)	7 (0)	12.5 (1.91)	< 6	< 6	< 6	< 6	< 6	< 6	**6.82**
SS11	6.88 (0.99)	< 6	16.5 (3.79)	< 6	< 6	< 6	< 6	< 6	< 6	< 6	< 6	< 6	7 (1)	< 6	< 6	**6.79**
	**6.97**	**8.88**	**7.56**	**7.37**	**7.43**	**6.43**	**9.19**	**9.53**	**7.01**	**8.56**	**9.83**	**7.44**	**6.80**	**8.17**	**8.49**	

*Neutralised cell-free supernatant after filtration with pH = 7 by addition of NaOH. More than 20 mm was considered strong inhibition (3+), between 11 and 20 mm was considered intermediate inhibition (2+) and less than 10 mm was considered low inhibition (+). The diameter of the colony is included. If no zone of inhibition was detected the result was reported as < 6 mm.

AV_{ZOIpa} means average zone of inhibition of all probiotics against the individual pathogen.
AV_{ZOIpro} means average zone of inhibition of individual probiotic against all pathogens.
